# Folate-Dependent Cognitive Impairment Associated With Specific Gene Networks in the Adult Mouse Hippocampus

**DOI:** 10.3389/fnut.2020.574730

**Published:** 2020-11-12

**Authors:** Abigail Lawton, Caroline R. Morgan, Caleb R. Schreiner, Chris G. Schreiner, Jacqueline Baumann, Britton Upchurch, Feifan Xu, Michael S. Price, Gary D. Isaacs

**Affiliations:** ^1^Department of Biology and Chemistry, Liberty University, Lynchburg, VA, United States; ^2^Department of Molecular and Cellular Science, Liberty University College of Osteopathic Medicine, Lynchburg, VA, United States; ^3^Department of Medicine, Duke University, Durham, NC, United States

**Keywords:** folate, cognition, hippocampus, gene expression, memory

## Abstract

Short-term folate deficiency has been linked to cognitive defects. Given folate's role in regulating nucleotide synthesis and DNA and histone methylation, these changes are often linked to altered gene expression and might be controlled by specific regulatory networks. In our study we examined the effects of folic acid (FA) deficient or replete diets in mice, containing either no source of folate or normal FA intake, beginning post-weaning and persisting through the end of adult life at 18 months. Our goal was to assess levels of cognition in these mice using the novel object test and then connect the cognitive results to genetic changes. FA deficient mice showed significant memory impairment compared to control counterparts beginning at 5 months and persisting through 17 months, as determined by the novel object test. These deficits were associated with 363 significantly downregulated and 101 significantly upregulated genes in the deficient condition compared to the control condition in microarray analysis of hippocampal tissue. Many of these gene expression changes were determined to be specific to the hippocampus. Significant ontological categories for differential genes included nucleotide regulation, ion channel activity, and MAPK signaling; while some of these categories contain genes previously mapped to cognitive decline, other genes have not previously been associated with cognition. To determine proteins possibly involved in regulation of these genes, we performed bioinformatics analysis and found enriched motifs of for MafB and Zfp410 binding sites. These genes and enriched motifs may represent targets for treatment or investigation of memory-related diseases.

## Introduction

Vitamin B9 can be found in a number of forms, referred to together as folates. The two most common folates are 5-methyltetrahydrofolate (5MTHF), the biologically active form found naturally in food, and folic acid (FA), the synthetic form used in supplements and food fortification (1). Folates are important one-carbon carriers in a number a cellular reactions including nucleic acid metabolism, amino acid metabolism, maintenance of DNA stability, and production of S-adenosylmethionine (SAM) for methylation of nucleic acids, neurotransmitters, phospholipids, histones, and other proteins (2–5). Gene methylation and expression often have an inverse relationship with decreased methylation resulting in increased expression (6, 7). Therefore, maintenance of proper folate levels helps avoid aberrant DNA methylation patterns, thus ensuring normal transcriptional regulation (8). Similarly, decreased folate levels can be associated with altered nucleic acid metabolism and altered gene expression (9). As such, folate deficiency has been associated with a number of diseases including various cancers, cardiovascular disease, and cognitive defects (10–18). Despite mandatory folate fortification in grains in many Western cultures, a variety of circumstances can lead to chronic folate deficiency including poor diet, chronic smoking, chronic alcoholism, intestinal diseases, medications, and gene polymorphisms (19–22). Although many of these habits and diseases may lead to chronic folate deficiency for the duration of adult life, little is known, regarding the effects of this deficiency throughout adulthood. Further, most studies on folate deficiency induce the deficiency early in life, either in *utero* or during weaning (12, 23). Although valuable, these studies provide no understanding of the many populations who receive adequate folate early in life but become deficient during adulthood. As such, it has yet to be determined to what extent adequate folate intake during early life can protect against folate deficiency later in life, despite the fact that this scenario likely affects many people.

Evidence has accumulated in recent years linking folate deficiency at many stages of life to neurological deficits, with the largest investigative emphasis on early life since the discovery of the critical role of folate in neural tube development (24–26). Folate deficiency in rodent pups during weaning has been linked to expression changes in genes associated with DNA methyltransferases (DMNTs) in the rat hippocampus and a reduced number of proliferating cells in the mouse hippocampus (27). This observed alteration in hippocampus structure due to folate deficiency holds true in the adult hippocampus as well; dietary folate deficiency during early adult life in mice reduces the number of proliferating cells in the hippocampus (28). Further, consistent with clinical observations that many dementia patients present with low plasma folate levels (29). A study of elderly Swedish patients receiving no folate or vitamin B12 supplementation determined that low serum levels of folate or B12 are indicative of a nearly doubled the risk of developing dementia compared to patients with normal folate or B12 levels (30). However, many of the molecular mechanisms underlying this relationship have yet to be elucidated.

While it is well established that folate deficiency during early life results in cognitive impairments (12, 13, 16), it is not clear whether adequate folate during major developmental milestones followed by inadequate folate in later life will exhibit the same effect. Folates play essential roles in both methylation and nucleic acid metabolism; therefore, we hypothesize that chronic post-weaning folate deficiency will result in expression changes in many additional hippocampal genes beyond DMNTs and that those genes may be linked to cognitive deficits. Because of the chronic nature of many conditions leading to folate deficiency in adult life, we investigated the effects of folate deficiency beginning post-weaning and extending through the duration of the adult mouse's life, in order to shed light on additional therapeutic avenues for treatment and prevention of cognitive decline.

## Methods

### Mice

**Figure 1:**
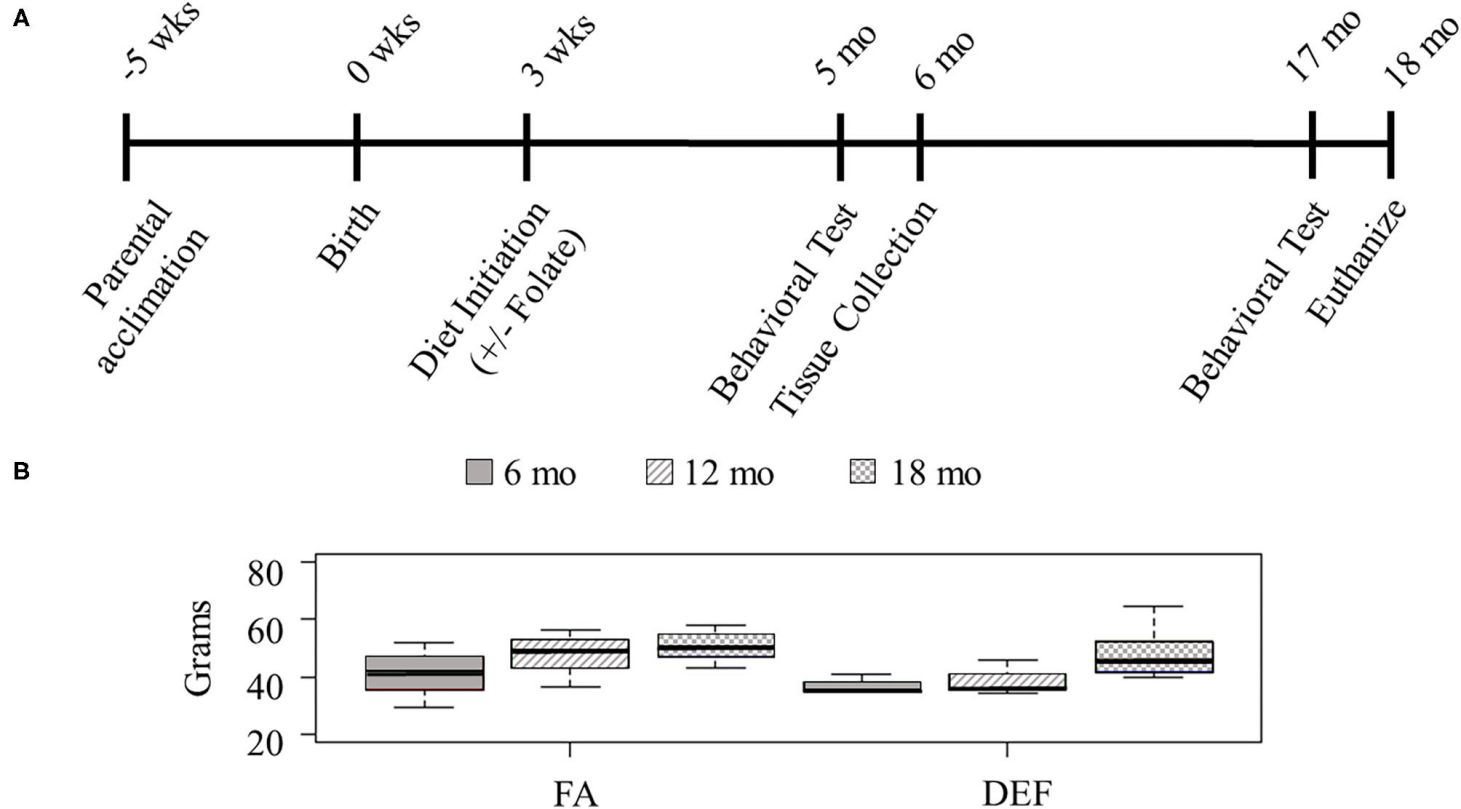
Study design. **(A)** Outbred 7-week-old CD1 mice were acclimated for this study with the introduction of a custom vitamin B deficient chow containing 1% succinylsulfathiazole and supplemented with B6, B9, B12 for 5 weeks prior to the birth of their pups. Mothers and pups were kept on this diet during weaning. After weaning (3 weeks post birth), the pups of half of the litters remained on the parental diet, and the pups of the other half of the litters were put on a folate-deficient diet. Behavioral tests were administered 5 and 17 months after birth, and tissues were collected following the completion of behavioral tests at each time point (about 1 month after their initiation). Results from littermates were averaged and considered to be *N* =1. **(B)** Mice were weighed throughout the course of the study to determine if diet had any effect on overall size of the mouse (*N* ≥ 3 for all ages and diets). Although all mice gained weight with age, there was no statistical difference between the various dietary conditions at any single time point (one-tailed homoscedastic student *t*-test: 6 months FA vs. DEF *P* = 0.29, 12 months FA vs. DEF *P* = 0.14, 18 months FA vs. DEF *P* = 0.36).

Twelve female outbred CD-1 mice (Charles River Laboratories, Wilmington, MA) were crossed with males of the same strain to produce pups for this study. This strain was chosen for the high genetic diversity, healthy offspring, and large litter size associated with outbred strains. Two weeks prior to breeding the mice were placed on a custom chow supplemented with FA (Figure 1A). Parental mice were approximately 42 days old at the time of breeding. Female pups were used for this study and remained on the parental diet with their mothers until they had been weaned. Female mice were used exclusively for this study due to housing constraints, at a limit of 5 females per cage. Once the pups were weaned, half of each litter continued on the parental diet while the other half began a FA-deficient diet. Each dietary condition contained three independent litters with multiple pups. At least one mouse from each litter at 6 and 18 months was euthanized by carbon dioxide asphyxiation, and all efforts were made to minimize suffering. Experimental protocols were approved by the Institutional Animal Care and Use Committee (IACUC) at Liberty University (protocol 3.160309). Mice were kept on a 12 h light/dark cycle in a temperature and moisture controlled room for the duration of the study.

### Diets

Parental mice were given a custom vitamin B-deficient chow (Envigo-Teklad Diets, Madison, WI; Supplementary Table 1) containing 1% succinylsulfathiazole to inhibit microbial folate production in the gut. Vitamins B_6_ and B_12_ were supplemented (35 μg/mL and 125 μ g/mL, respectively, Solgar, Leonia, NJ) dissolved in drinking water for all mice since the custom chow lacked these two B Vitamins and our study wanted to determine the effects of folate (B_9_) only. FA supplemented mice were given water also fortified with FA (10 μg/mL), following normal dietary recommendations (31). Initial calculations of average daily water consumption was approximately 5 mL per day per animal and determined the supplemental vitamin concentrations in the water (Supplementary Figure 1). Specifically, water bottles were filled and weighed before placing them in the cage. Twenty-four hours later bottles were removed and weighed again to determine the water loss due to animals drinking and bottles dripping. This procedure was conducted twice for 12 cages containing no more than two mice each. In order to determine the amount of water loss due solely to bottles dripping, 11 twenty-four hour trials were conducted in cages without mice. It was determined 2.7 + 0.1 mL (SEM) was lost due to water dripping. The average daily water consumption per mouse per day for all 12 cages was calculated as the daily change in water in each cage minus the daily average loss due to dripping divided by the number of mice in the cage, which was calculated to be 5.6 + 0.4 mL (SEM). Averages and error bars were calculated in Microsoft Excel using standard error of the mean. Fortified water was made fresh and changed every 3 days. This method of folate supplementation has been previously reported in mice (32–35). Serum folate levels were not measured since significant reduction in serum folate has been observed following shorter periods of folate deficiency (36). Further, the aim of this study was to assess the stress response of a chronically folate deficient diet, whether or not the response is directly linked to depleted serum folate. Mice were weighed at 3 stage of adult life (6, 12, and 18 months) to ensure that diets did not affect overall health; average weights and standard error for each dietary condition at each time point were analyzed using R studio version 3.1.0.

### Behavioral Tests

Novel object tests were performed on at least three mice representing independent litters for each of the dietary groups at 5 and 17 months, allowing for 1 month of cognitive testing prior to euthanization at each time point. Each test was composed of a 5-min interaction with two identical objects followed by a second 5-min interaction 24 h later with one of the objects replaced with a new object. Two stopwatches recorded the amount of time spent with the two objects during this second trial. Data was recorded as the percentage of time spent investigating the new object relative to the total time investigating both objects. Familiar and novel object sets were different for each time point tested to preserve the integrity of the test and provide multiple object pairs testing reliability. At 5 months the familiar objects were two T25 cell culture flasks filled with blue-colored water and the novel object was a green Lego object of similar shape and size. At 17 months the familiar objects were two green turtles made of rubber and the novel object was a blue dolphin of similar size and material. Results were analyzed using a two-tailed homoscedastic student *t*-test at the 0.05 level comparing deficient to control diets at each time point using Microsoft Excel.

### Tissue Extraction and RNA Isolation

After the completion of behavioral studies at 6 months, mice were sacrificed to obtain tissue samples for both dietary groups (FA and DEF). After isolation of whole brain samples, a sagittal cut divided the hemispheres, and the samples were placed in RNALater (Applied Biosystems, Foster City, CA) for 10 min to preserve RNA and dehydrate the tissue. The hippocampal dissection procedure followed a previously described account (https://www.youtube.com/watch?v=tdEvicXkMCk, permission was obtained from the author for distribution of the video). Isolated hippocampi were soaked in RNALater for 24 h at 4°C before storage at −80°C.

RNA was extracted from hippocampal tissue using Trizol (Invitrogen) according to the manufacturer's instructions. Briefly, tissue was homogenized in 0.1 × Trizol volume and incubated at room temperature for 5 min. Chloroform was added up to 0.2 × Trizol volume, and samples were incubated an additional 2 min at room temperature before being centrifuged at 12,000 × *g* at 4°C for 15 min. The aqueous phase was separated, mixed with 0.5 × volume of isopropanol, incubated for 10 min at room temperature, then centrifuged at 12,000 × *g* at 4°C for 10 min. The pellet was resuspended in 75% ethanol, vortexed, and centrifuged at 12,000 × *g* at 4°C for 5 min. Finally, the sample was dried and resuspended in 30 μL of RNase-free water and incubated at 55°C for 10 min. The sample was quantified and purity was assessed using a Nanodrop ND-1000 spectrophotometer (Thermo Fisher Scientific, Waltham, MA).

### Microarray Analysis

The sample preparation and microarray hybridization were performed based on the manufacturer's standard protocols. Purified RNAs for each dietary condition at 6 months (*N* = 3) were submitted to Arraystar for microarray analysis using the V3.0 Mouse LncRNA Array. These replicates fell within Arraystar's suggested parameters of 3–6 samples per group for detection of expression changes with an effect size of 2, false discovery rate of 0.05, and statistical power of 0.8. A total of 35,923 lncRNAs and 24,881 mRNAs were used to assess the transcriptomes of these samples. A total of 5,000 ng of RNA was provided at a concentration of 200 ng/μL. RNA integrity and quantity were verified using standard denaturing agarose gel electrophoresis and the NanoDrop ND-1000. For microarray analysis, Agilent Array platform was employed, and results were normalized by Lowess normalization using R version 3.1.0 (R Foundation for Statistical Computing, Vienna, Austria) and the package “ggplot2” for images. Quantile normalization and subsequent data processing were performed using the GeneSpring GX v12.1 software package (Agilent Technologies). After quantile normalization of the raw intensity data, the differentially expressed genes between the comparison groups were identified by fold change (FC ≥ 2.0) and by statistical significance (*P* ≤ 0.05 by unpaired, two-tailed *t*-test) (37). This data is available at the NCBI gene expression omnibus under accession GSE148126.

### Quantitative PCR

Gene-specific quantitative PCR (qPCR) confirmations were used to validate the microarray data. A High-Capacity RNA-to-cDNA Kit (Applied BioSystems, Foster City, CA) was used to convert RNA to cDNA according to manufacturer's instructions. Briefly, 2 μg of RNA was incubated for 60 min in a 30 μL reaction containing 15 μL of 2 × RT Buffer mix and 1.5 μL of 20 × RT enzyme mix brought up with nuclease-free water. The reaction was stopped by heating to 95°C for 5 min. The cDNA was diluted 1:100 with nuclease-free water for use in qPCR reactions. Primers for qPCR were designed using the UCSC BLAT tool on Arraystar probes for significant differentially expressed genes from the microarray (https://genome.ucsc.edu/cgi-bin/hgBlat). This tool allowed us to map all primers to the same transcript as the one targeted by Arraystar probes by designing primers to contain the probes region when possible or to be within the same exon as the probe if the exact probe region was not possible. Primer3 was then used to design primers based on the genetic regions determined in BLAT (http://bioinfo.ut.ee/primer3-0.4.0/primer3/). Parameters for Primer3 software were as follows: primer T_m_ was 59 ± 2°C, primer size was 20 ± 2 bp, and GC clamps were used when possible; all other criteria were left as the default software settings. Primer sequences are given in Supplementary Table 2. The 25 μL qPCR reactions contained 0.02 μg of cDNA, 12.5 μL of 2 × SybrGreen PCR Supermix, and forward and reverse primers with a final concentration of 0.625 μM each. All reactions were performed in duplicate for each cDNA pool (*N* = 3 independent biological replicates for each condition) using *Gapdh* as the control gene; reactions lacking cDNA template were included as controls for primer self-annealing and amplification. Amplification was performed using a BioRad MJ Mini Personal Thermal Cycler. The qPCR cycling parameters were as follows: 95°C for 3 min (1 cycle), 95°C for 10 s followed by 59°C for 1 min (40 cycles), finished with a melt curve analysis. The amplification graphs were generated using BioRad CFX manager 2.0. The quantification cycle (Cq) values were obtained for all samples and used for quantification with the 2ΔCq method with one-tailed equal variance *t*-tests and standard error of the mean using Microsoft Excel (38).

### Gene Ontology

Gene ontology was performed on significantly differentially expressed genes from microarray analysis using GeneCodis, a gene annotation website (39–41). Two random gene lists were also analyzed using GeneCodis and compared to the list of differentially expressed genes. The following criteria were used to determine significant single enrichment gene ontologies: GeneCodis level 7 (most stringent), minimum of 28 genes associated with the ontology, Chi-square value greater than Chi-square from any random list. Additionally, the following criteria were used to determine significant pathway enrichment gene ontologies: GeneCodis level 7, at least twice as many genes as any random list, Chi-square value greater than Chi-square from any random list. The enrichment value was calculated in Microsoft Excel using the following formula:

$$\begin{array}{l} \frac{\frac{Number\;of\;annotated\;genes\;in\;input\;list}{Number\;of\;genes\;in\;reference\;list}}{\frac{Total\;number\;of\;genes\;in\;input\;list}{Total\;number\;of\;genes\;in\;reference\;list}} \end{array}$$

where the input list consisted of all significantly expressed genes from microarray analysis (with separate input lists for upregulated and downregulated genes) and the reference list consisted of all expressed genes on the microarray.

### Motif Analysis

A publicly available motif discovery tool called Hypergeometric Optimization of Motif EnRichment (HOMER) was used to identify transcription factor motifs within the target genes regulated by FA (42). In this analysis, two files were run separately: one containing the up regulated genes and one containing the down regulated genes from the RNA microarray results of the FA diets at 6 months. The HOMER script findMotifs.pl was used for the genome of “mouse.” Parameters included a starting point of 2,000 bases downstream of the TSS and 500 upstream were specified with a background list of all the genes from the microarray test. Results consisted of *de novo* and known motif sequences generated by position weight matrixes with their *P*-value, percent of the target and background, and the best match of the protein that HOMER found based on their database from UCSC.

## Results

### Post-weaning Effects of Folate on Growth

Since little is known about long-term effects of folate deficiency, we designed a study to follow folate deficient mice from infancy through late adulthood (Figure 1A). Cohorts of 3 FA deficient and 3 FA control mice were followed for each folate source. Since mice remained on their designated diet for the duration of their life, we weighed them at 3 stages of their adult life to ensure the diet did not affect their overall health (6, 12, and 18 months—representing young adult, middle-aged adult, and senior adult). Although all mice in each diet gained weight as they aged, at any given time point there was no significant difference between the weights of mice on a folate diet compared to mice on a folate deficient diet (Figure 1B, *P* ≥ 0.6 in all conditions).

### Effects of Folate Restriction on Cognition

**Figure 2:**
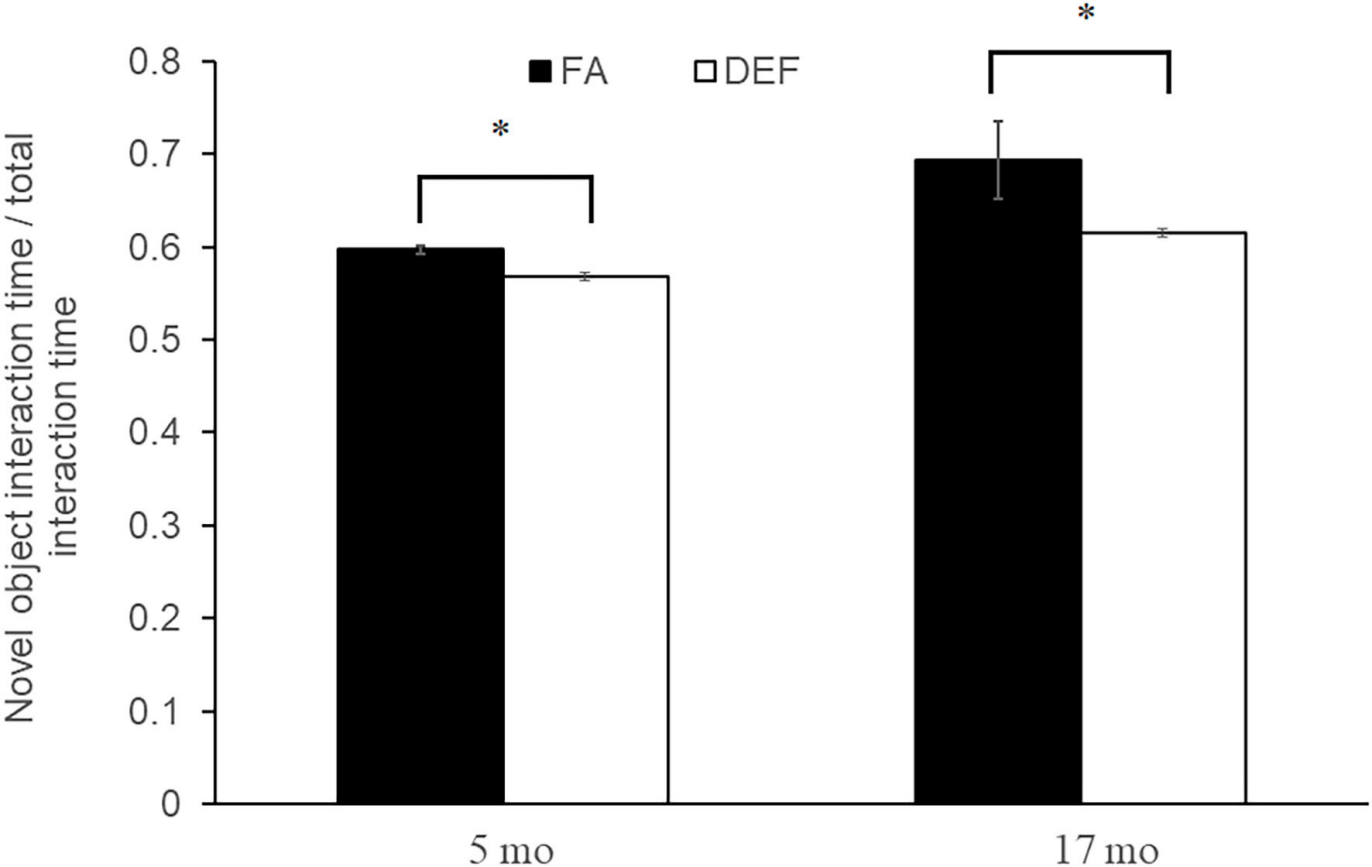
Novel object test for object memory. A novel object test was used to assess memory in each dietary condition by comparing percent time the mouse spends with a new object vs. an old object. Three litters of mice were used per condition with multiple litter mates averaged to represent one data point. Five-month-old folic acid (FA) litters contained 4 mice each and FA-deficient (DEF) litters contained 3 mice each. Each 17-month-old litter contained 3 mice. All comparisons represent *P* ≤ 0.03 (noted by astrisks). Error bars represent SEM values calculated from the independent biological replicates across all litters.

The novel object test for memory was administered to mice on both diets to assess cognitive abilities associated with each diet. Although the weights of the mice were not affected by dietary condition, a significant difference was noted in cognitive abilities beginning at 5 months (young adult) and persisting through 17 months (old adult). Mice on the FA deficient diet spent significantly less time with the novel object (60 and 69% of time with novel object in control mice compared to 47 and 53% of time in deficient mice at 5 and 17 months, respectively; *P* < 0.05 for all comparisons) compared to mice on the FA control diet, demonstrating decreased memory capabilities as assayed over a 24-h period (Figure 2). Even though cognitive deficit persisted through 17 months, the initial observance at 5 months suggests that the effects of folate deficiency maybe be established at this early time. For this reason, we focused our transcriptional analysis on mice collected immediately following the 5-month behavioral test.

### Microarray Analysis to Determine Folate-Regulated Genes

**Figure 3:**
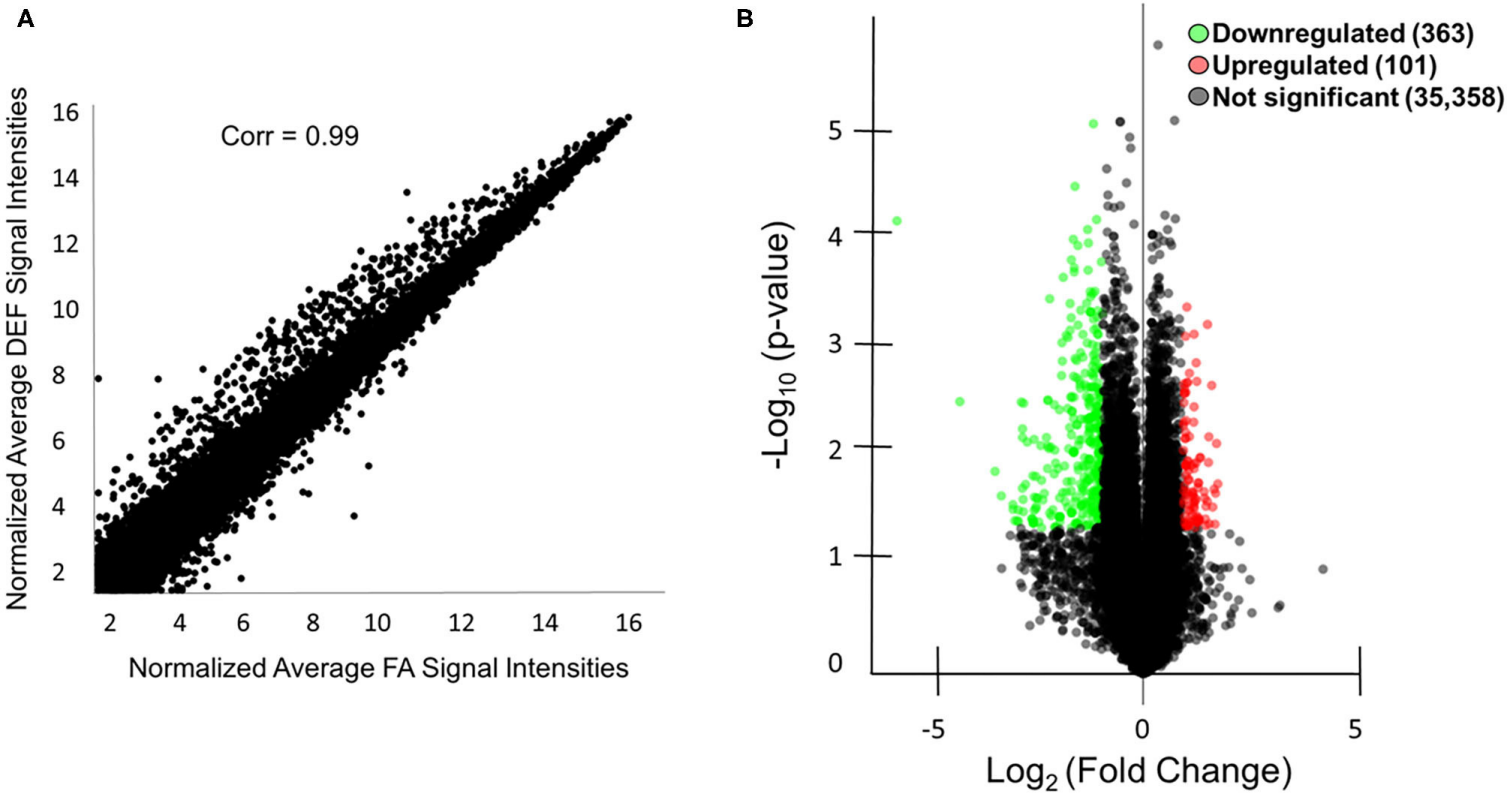
Microarray analysis of mice with and without FA. **(A)** A scatter plot is shown comparing the microarray data of the average normalized signal intensities for the three FA replicates vs. the average normalized signal intensities for the three deficient (DEF) replicates. The correlation coefficient was calculated using R version 3.1.0. Correlations between individual replicates within the FA group (0.99, 0.98, 0.98) and within the deficient group (0.98, 0.99, 0.98) were also calculated to show the similarities within each condition. **(B)** A total of 363 mRNA or lncRNA regions were downregulated (green) in the deficient condition compared to the FA condition, and a total of 101 mRNA and lncRNA regions were upregulated (red) in the same comparison as indicated by the volcano plot. An additional 35,358 mRNA and lncRNA regions were not differentially regulated (black). Genes were considered differentially regulated if they had fold change >2 and *P* < 0.05 by microarray analysis.

**Figure 4:**
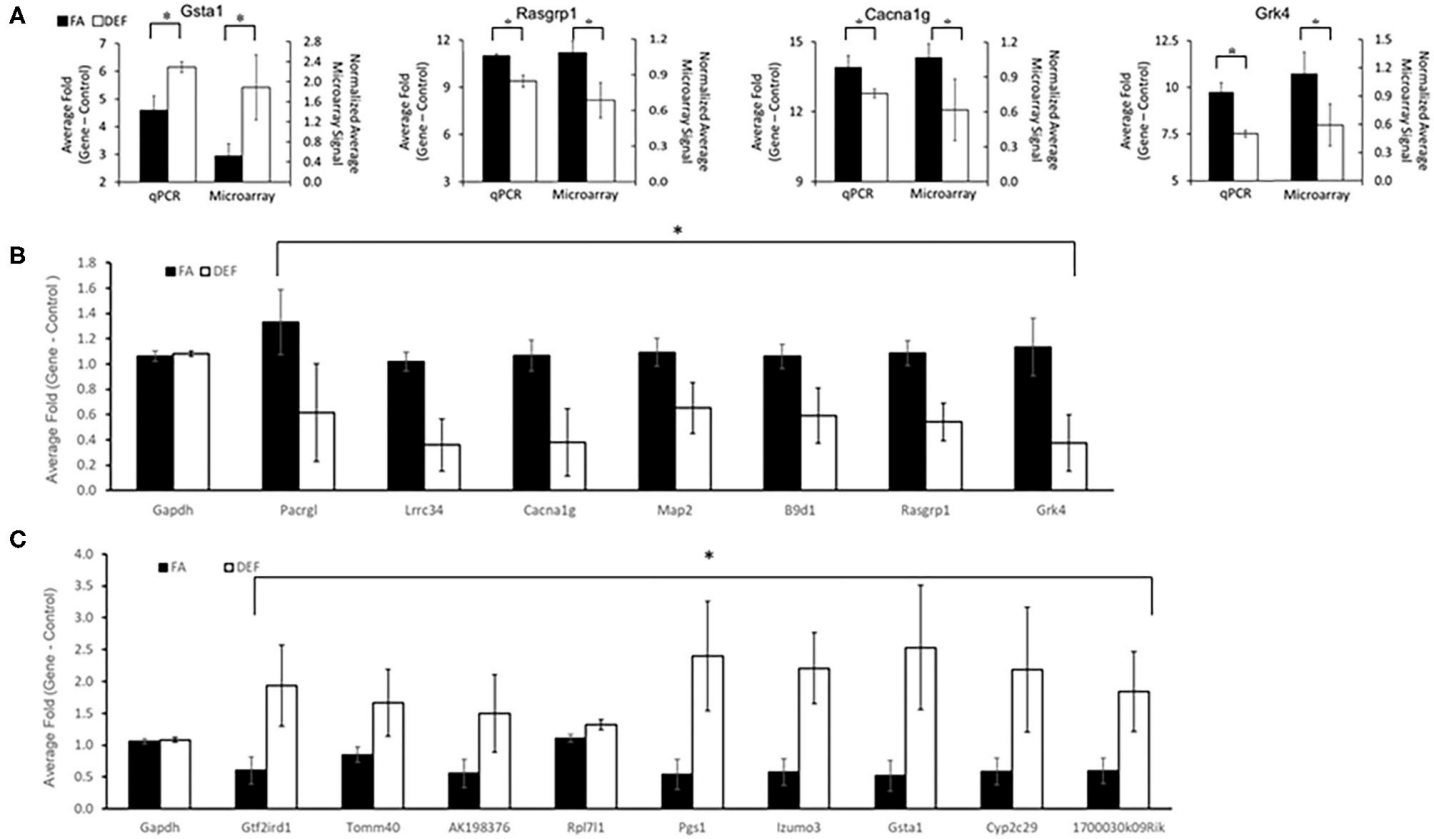
qPCR confirmations of specific genes shown to be differentially expressed in microarray data. A subset of genes considered to be significant (fold change ≥2 and *P* ≤ 0.05) by microarray analysis of the hippocampus were confirmed to be significant by qPCR (*P* ≤ 0.05). In addition one gene (Lrrc34) was chosen below the threshold (fold change 1.9). Genes were chosen for confirmation to represent a variety of *P*-values, fold changes, and ontologically significant genes. Primers were designed around the same region or within the same exon as the microarray probe when possible to ensure qPCR was targeting the same transcript as the microarray. Each gene was tested using at least *N* = 2 for independent biological samples for each dietary condition. **(A)** Four examples of microarray vs. qPCR comparisons of confirmed genes are shown. A number of genes were confirmed to be downregulated **(B)** or upregulated **(C)** in the deficient condition compared to the FA condition (*P* ≤ 0.05 for all comparisons as noted by astrisks). Each experimental gene was normalized to the average *Gapdh* expression for its respective dietary category. *Gapdh* expression was not significantly altered in any deficient vs. folate comparisons. Plotted *Gapdh* levels are relative to the expression of one FA *Gapdh* replicate to demonstrate the lack of variability between independent replicates and to demonstrate the lack of folate dependence. Error bars represent SEM values calculated from the three normalized array values or 3-fold values prepared from three independent biological replicates.

In order to characterize molecular mechanisms associated with the cognitive decline observed in mice who were chronically FA deficient, hippocampal gene expression in the FA deficient mice was compared to expression in FA control mice using RNA microarrays. These microarrays were obtained for the 6-month time point to assess primary genetic changes associated with the dietary conditions. These arrays found a high correlation of expressed transcripts among all RNA preps both within and between dietary groups reflecting the large similarity of the transcriptomes from these mice (Figure 3A, *r* = 0.99). We identified 363 transcripts to be significantly downregulated and 101 transcripts to be significantly upregulated (*P* < 0.05, fold change > 2) in the deficient condition compared to the FA condition (Figure 3B, Supplementary Table 3). A subset of 16 of these transcripts were confirmed by qPCR to be differentially regulated due to FA supplementation (Figures 4A–C, *P* ≤ 0.05).

### Folate-Dependent Genes Specific to Hippocampus

**Figure 5:**
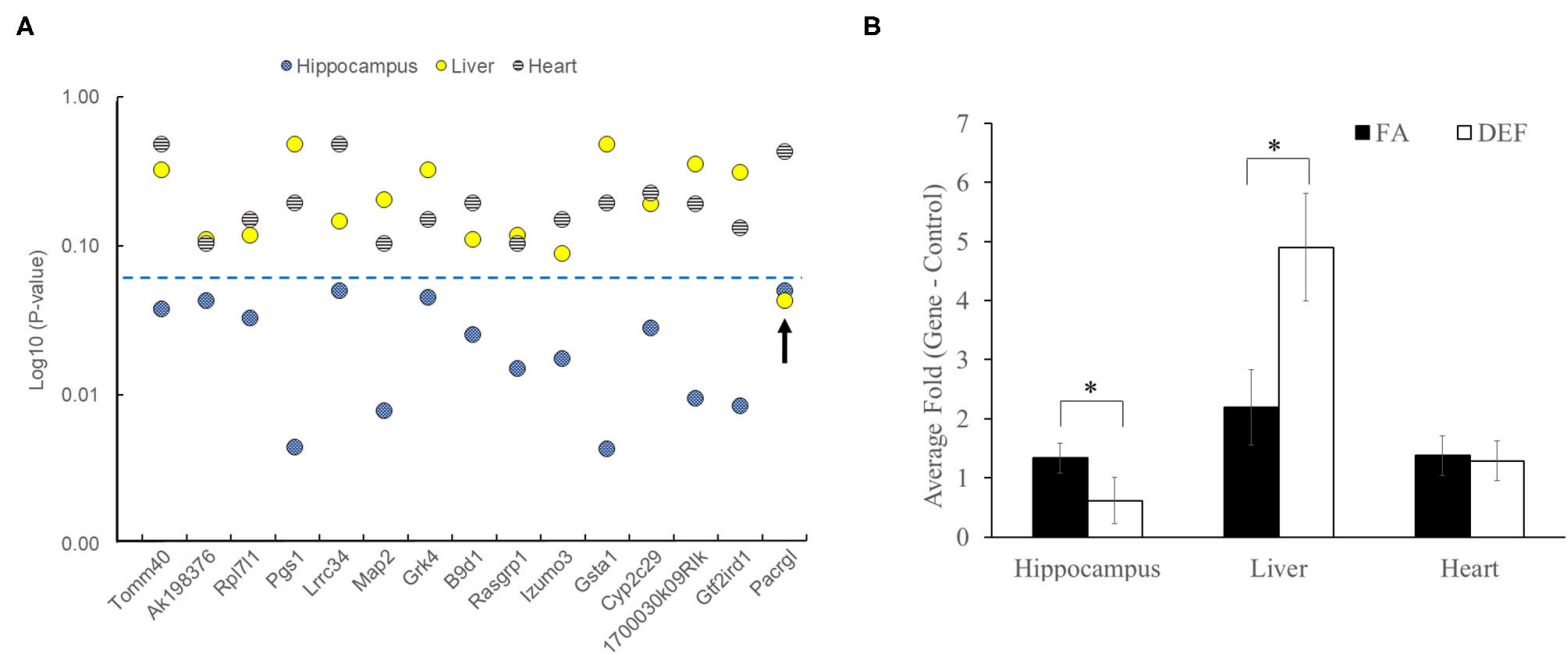
Folate-dependent genes are not ubiquitously expressed. Each of the genes confirmed to be differentially expressed in hippocampus was also tested in liver and heart tissue of the same mice to determine if differential expression was systemic or tissue specific. Each gene was tested on 3 independent biological replicates in the FA and deficient (DEF) conditions. **(A)** Of the 15 genes tested, only one was found to be differentially expressed in the liver (*Pacrgl*), and no genes were found to be differentially expressed in the heart. The blue dotted line represents a significant *P*-value (≤0.05). **(B)** Interestingly, only one of the differentially expressed hippocampus genes was also differentially expressed in the liver (*Pacrgl*, hippocampus *P* = 0.05, liver *P* = 0.04; significance noted by asterisks). Further, *Pacrgl* was significantly downregulated in the hippocampus and significantly upregulated in the liver in the deficient vs. folate comparison. This same gene was not differentially expressed in the heart (*P* = 0.43). Error bars represent SEM values calculated from the 3-fold values prepared from three independent biological replicates.

Since folate is known to have tissue-specific effects, we further investigated if any of our 16 confirmed differentially expressed genes were indeed specific for hippocampus (43, 44). We analyzed the same 16 transcripts using cDNA pools generated from liver and heart tissue from the same mice. Interestingly, none of these genes were differentially expressed in the heart, and only one (*Pacrgl, P* = 0.04) was differentially expressed in the liver (Figure 5A, Supplementary Figure 2). Further, we observed that this *Pacrgl* gene was significantly downregulated in the deficient hippocampus and significantly upregulated in the deficient liver (Figure 5B), an observation that is consistent with previous studies that noted the hippocampus and liver exhibit opposite genetic responses to short-term folate deficiency (45–48). Thus, it is likely that many of the differentially regulated genes found on the microarray are specific to the hippocampus and may be involved in the observed memory deficits found in these animals.

### Gene Ontology of Folate-Regulated Genes

To shed further light on the molecular mechanisms involved in our observed memory deficits precipitated by chronic FA deficiency, we performed gene ontology on the differentially regulated genes using GeneCodis (Table 1, Supplementary Table 4). The largest ontological category was the 47 downregulated genes associated with the regulation of nucleobase-containing compounds, which is not surprising considering the crucial role the folate cycle plays in nucleotide synthesis. Indeed, many of the other downregulated ontological categories involved DNA and RNA associated mechanisms. Perhaps a more interesting ontological category is the 11 downregulated genes associated with MAPK signaling pathway, a number of which (*Arrb2, Cacna1g, Fgfr4, Taok1, Cacna1h*) have been implicated in Alzheimer's disease (AD) (49–53). Additionally, two downregulated ontological categories involve ion channel activity, which has also recently been implicated in AD (54).

**Table 1:** Gene ontology of folate-regulated genes.

**Gene set**	**Gene ontology**	**Genes**	***P*-value**	**Enrichment**
Genes downregulated (317 genes)	Regulation of nucleobase-containing compounds	47 Genes	0.0489	1.44
	Transcription from RNA polymerase II promotor	22 Genes	0.0226	1.85
	Intracellular protein kinase cascade	18 Genes	0.00276	2.34
	Detection of stimulus in sensory perception	7 Genes	2.6 × 10^−5^	5.72
	Ion channel activity	9 Genes	0.0397	2.35
	Gated channel activity	8 Genes	0.0202	2.72
	Double stranded DNA binding	5 Genes	0.0312	3.22
	MAPK signaling pathway	11 Genes	0.000968	3.58
Genes upregulated (89 genes)	Heparin binding	3 Genes	2.41 × 10^−6^	10.6

*All genes that were differentially expressed in the deficient condition compared to the FA condition according to microarray analysis (fold change > 2 and P < 0.05) were analyzed with GeneCodis using all of the microarray probes as the background list. Two random lists of genes of the same size as the upregulated and two of the same size as the downregulated experimental lists were also run as controls for false positives in gene ontologies. Annotations are presented in this table if (a) the gene ontology represented a greater number of genes and a lower P-value than those resulting from a corresponding random list and (b) the annotations appeared to be relevant to this study. P-values were calculated by GeneCodis using Chi-square tests*.

### Motif Analysis

**Figure 6:**
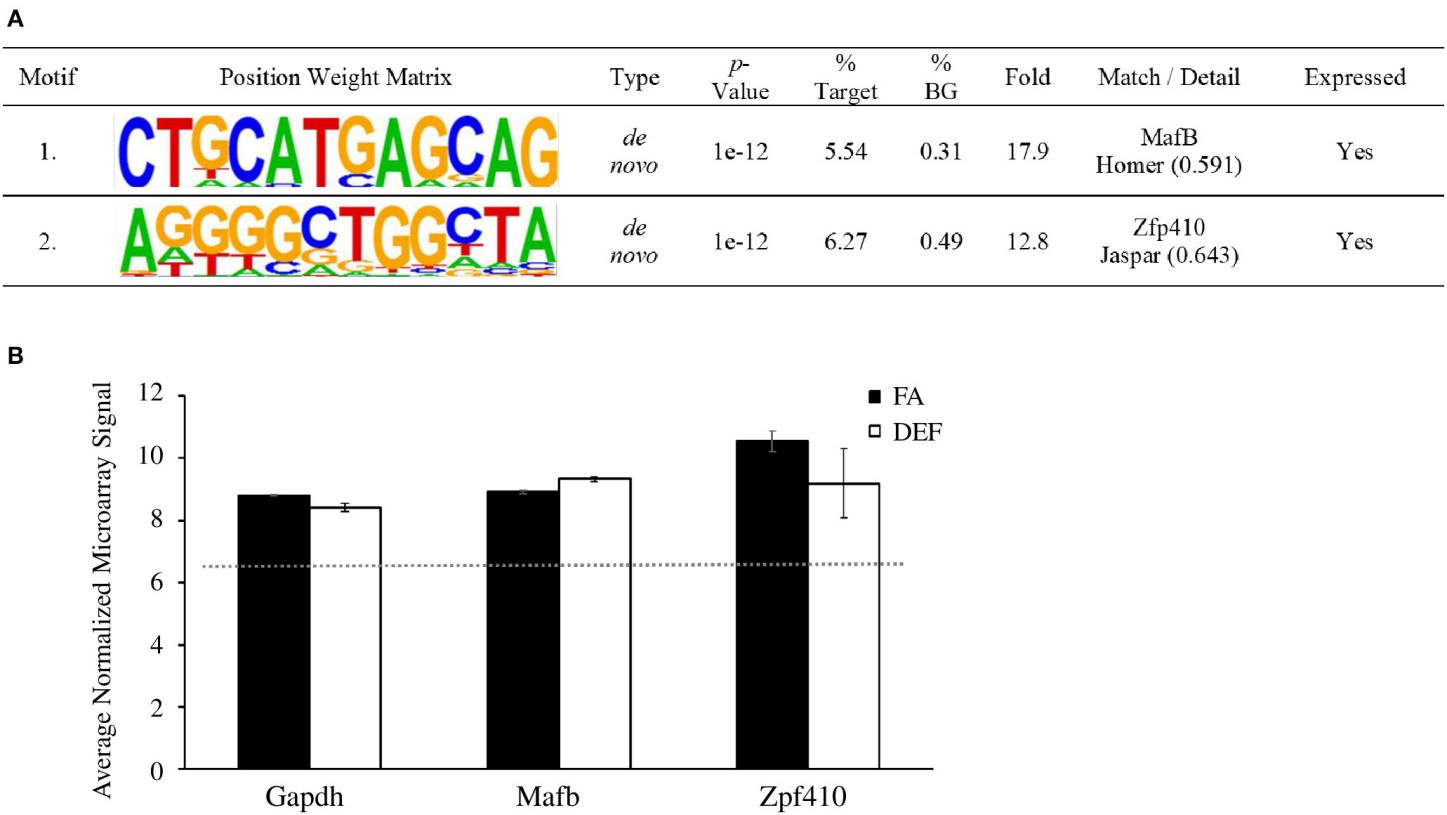
Sequence motifs enriched in folate-regulated genes. **(A)** Differentially expressed RNA was analyzed with HOMER to determine significant transcription factors associated with differentially regulated genes. A target list of 224 sequences was compared to 19,048 background sequences. Transcription factor binding sites were mapped to be −2000 or +500 bp relative to the TSS of differentially expressed microarray genes. This table shows a list of motifs and their respective transcription factor that were enriched in the deficient condition compared to the FA condition and associated with each motif. **(B)** HOMER analysis showed two significantly enriched motifs that match the binding sequence for *Mafb* and *Zfp410*, respectively. The average normalized microarray levels (log2 values) for these genes are shown. *Mafb* and *Gapdh* are represented by one probe each while *Zfp410* (which has three probes on the array) is represented by the probe with the highest expression level. The expression of *Gapdh* is a reference along with the median normalized value of all transcripts on the array (red dotted line). Error bars represent SEM values calculated from the three normalized array values prepared from three independent biological replicates.

Since altered expression implies altered gene regulation, we then examined the promoter sequences of folate-regulated genes in order to determine if any transcription factor binding sites were enriched in a diet-dependent manner. We used the motif discovery tool HOMER to determine significantly enriched motifs associated with differentially regulated transcripts from microarray analysis in the deficient mice compared to the FA mice. To do this we compared promoter sequences (−2000 bp to +500 bp) of the differentially regulated genes searching for both known and *de novo* motifs. HOMER determined 2 significantly enriched motifs (*P* ≤ 0.0001) as *de novo* motifs that match previously identified binding sites for MafB and Zfp410 (Figure 6A). This finding is consistent with experiments demonstrating a potential neuroplasticity role for *Zfp410* in rat hippocampal dendrites (55). We confirmed that *MafB* and *Zfp410* are indeed expressed in the hippocampal transcriptome using our microarray data (Figure 6B).

## Discussion

We demonstrated that chronic post-weaning folate deficiency in mice produced memory deficits observed in early adulthood and in old age. Although the mice received adequate folate during major developmental milestones, this early life folate was not sufficient to protect against detrimental outcomes of late life folate deficiency. Since the hippocampus is largely responsible for memory consolidation, we mapped differences in hippocampal gene expression between FA control and FA deficient mice and found a large number of genes to be differentially expressed in the FA deficient group. We believe that many of these genes may have implications in memory functions (Table 1, Supplementary Table 4).

The MAPK signaling pathway findings from our gene ontology of downregulated genes present an interesting category of potential genes to target for memory-related diseases, especially given that half of our genes in this category are already implicated in AD (Supplementary Table 3) (49–53). Specifically, *Arrb2* polymorphisms are indicated as risk factors for late onset AD; *Taok1* has been shown to actively phosphorylate tau in Alzheimer's brains; folate receptor α, which binds to promotor regions of *Fgfr4*, showed increased expression in AD fibroblasts; *Cacna1g* is downregulated in AD brains; and *Cacna1h* is downregulated in AD human neurons. MAPK signaling in general has been implicated in AD, so the additional genes noted in our ontology list may present novel specific targets for Alzheimer's investigations (56).

Similarly, ion channels have been generally implicated in AD, so our ion-channel ontological category genes may present specific targets for nutrition-related cognitive decline (54). *Mcoln1* (a.k.a. *Trpml*) is implicated in Alzheimer's pathology through dysregulation of autophagy (57). *Trpml* was shown to be downregulated in a mouse model of AD (58) and downregulated in our folate deficient/cognitively impaired mice (2.4-fold down, *P* = 0.001; Supplementary Table 3). Additionally, a growing body of evidence has implicated *Tomm40* polymorphisms as predictors of late onset AD (59–61) The extended regulatory region of TOMM40 in humans, which includes APOE and APOC2, has recently been reported to be hypomethylated in AD subjects correlated with its increased expression in AD (62). We have demonstrated that folate-deficient mice which exhibit cognitive impairment also have an increase in *Tomm40* expression further implicating this gene. *Grid1* expression in human females is positively correlated with protection from AD risk (63). Our study with cognitively impaired female mice demonstrated a downregulation of Grid1 (2.9-fold down, *P* < 0.001; Supplementary Table 3) which also suggests the positive correlation of *Grid1* expression and cognitive health. It is interesting to note that this previous study also demonstrated *Grid1* expression was associated with AD risk in male mice. Although our study did not include male mice, future investigation would be warranted to determine if folate deficiency was associated with an opposite transcriptional response (i.e., upregulation of *Grid1*) in those animals. Our data also indicates that *Vdac2* expression is positively correlated with cognitive health as it is downregulated in the folate-deficient mice (2.3-fold down; *P* = 0.035; Supplementary Table 3) which is consistent with lower Vdac2 protein levels in the brains of AD patients (64) yet were shown to be elevated in the brains of an AD mouse model (65, 66). Regarding *Vdac2*, we are unable to resolve this contradiction between these two models although our results using folate restriction in a non-transgenic mouse may more accurately model the genetic changes occurring in cognitive decline in humans.

Further, altered sensory perception is associated with AD (67, 68). Specifically, olfaction deficits have long been associated with AD (69, 70) and even used in some cases as a diagnostic factor (71). Additionally, expression of a number of olfactory receptors is altered in the cortex and hippocampus of AD mice, and olfactory receptors are found near amyloid plaques (72). Genome wide association studies have implicated *Col11a1* in AD, as this gene was downregulated in the hippocampus of Alzheimer's patients (58). Thus, our data is consistent with prior studies linking gene regulation differences with cognitive decline.

Knowing that folate is relevant in regulating nucleotide synthesis and methylation patterns in all tissue throughout the body, we compared expression of a subset of our significant hippocampal genes to expression in two other body tissues: heart and liver. None of the genes we tested were significantly different in the heart and only one was differentially expressed in the liver, demonstrating tissue-specific effects of folate deficiency. Further, the one gene (*Pacrgl*) that was differential expressed in the liver showed an opposing expression pattern between the two tissues, being downregulated in the deficient hippocampus and upregulated in the deficient liver. This is consistent with short-term folate deficiency studies that demonstrate opposite outcomes of folate deficiency in the liver vs. the hippocampus (46, 47, 73). This opposing expression pattern together with a large lack of long-term studies examining gene-specific outcomes of folate deficiency, indicate the need for future studies investigating the effects of chronic folate deficiency on other tissues. This is particularly true for both the liver due to its central role in folate metabolism and the heart due to the documented association between folate deficiency and cardiovascular disease (74, 75). Additionally, our tissue-specific findings demonstrate the likelihood that many of the genes we found to be statistically significant in the folate deficient hippocampus are likely associated with our observed cognitive outcomes and may serve as targets for treatment and prevention of memory-related diseases such as Alzheimer's and dementia.

All of our ontological categories containing genes with known cognitive implications represent genes downregulated in the folate deficient condition; indeed, we found that of all of our differentially expressed genes, over three times more genes were downregulated than upregulated. This may initially seem counterintuitive since folate is an important methyl donor, and decreased DNA methylation often leads to increased gene expression (6, 7). Indeed, this inverse relationship between methylation and expression has been shown to hold true in a folate-deficient liver, with overall global hypomethylation observed in mice on folate deficient diets (46). However, while global hypomethylation is often an outcome of folate deficiency, site-specific hypermethylation also occurs (73). Further, folate deficiency may result in different outcomes in different tissue types; the rat liver and brain have been shown to react to folate deficiency in opposing manners with brains exhibiting global hypermethylation while livers exhibit global hypomethylation in response to folate deficiency (26).

Our bioinformatics analysis of differentially expressed genes implicates factors that may influence the gene expression changes due to folate deficiency. We observed enriched motifs of binding sites for two proteins, MafB and Zfp410 (Figure 6). Although expression levels of these two genes are not significantly different between dietary conditions by microarray analysis, they may play in important role in regulating a number of genes whose expression was observed to be significant in the FA deficient condition. Perhaps folate's role in altering the hippocampal expression landscape can be partially explained by post-translational modification of MafB and Zfp410 or by altered methylation status in their binding regions. This is consistent not only with folate's pivotal role in DNA methylation, but also with the implications of folate deficiency on post-translational modification of various proteins including histones, septins regulating neural tube closure, and endothelial nitric oxide synthase in cardiovascular disease (76–78).

It is important to note that although these genes changed expression in response to inadequate folate intake, the exact mechanisms by which these genes changed were not determined in this study. Furthermore, observed gene expression differences at 6 months that correlate with early cognitive deficits may not persist at later time points (18 months). It is tempting to assume that since folate plays a major role in DNA methylation, which in turns plays a major role in gene expression, the genes may exhibit altered expression patterns due to altered methylation status. However, methylation status was not determined in this study, nor were levels of folate or SAM in the blood and hippocampus; therefore, we cannot rule out alternative mechanisms leading to the observed behavioral deficits and associated alterations in gene expression. Moreover, thus study used female pups due to housing constraints. Additional studies using male mice would need to be conducted to evaluate the potential of sex bias in our data. Taken together, data presented here emphasizes the importance of folate supplementation for the prevention of cognitive decline in adolescence and early adulthood. This is of particular importance to at risk categories such as malnutrition, substance abuse, and alcoholism.

In summary, we showed that folate deficiency beginning post-weaning and persisting through late adulthood induced memory deficits which can be linked to expression changes in over 400 genes, most of which were downregulated in the FA deficient condition. While many of these genes are expectedly related to regulation of nucleotide-related functions, a significant number are also associated with MAPK signaling and ion channel activity. Further, binding sites for two proteins MafB and Zfp410 were enriched in the FA deficient condition, perhaps representing regulatory pathways altered by folate deficiency. These genes and motifs may present novel targets for treatment of memory-related diseases such as AD.

## Data Availability Statement

The datasets presented in this study can be found in online repositories. The names of the repository/repositories and accession number(s) can be found below: https://www.ncbi.nlm.nih.gov/, GSE148126.

## Ethics Statement

The animal study was reviewed and approved by Institutional Animal Care and Use Committee (IACUC) at Liberty University.

## Author Contributions

GI: project conception, development of overall research plan, study oversight, and final decision for publication. AL: extraction of liver and heart RNA, microarray analysis, qPCR confirmations and comparisons, and paper writing. CRS, CGS, and CM: animal care, data collection for behavioral tests, extraction of hippocampal RNA, and production of hippocampal cDNA for microarray. JB and MP: bioinformatics. BU and FX: gene ontology. All authors have reviewed and approved the manuscript.

## Conflict of Interest

The authors declare that the research was conducted in the absence of any commercial or financial relationships that could be construed as a potential conflict of interest.

## Abbreviations

5MTHF: 5-methyltetrahydrofolate
AD: Alzheimer's disease
DNMTs: DNA methyltransferases
FA: folic acid
HOMER: Hypergeometric Optimization of Motif EnRichment
qPCR: quantitative PCR
SAM: S-adenosylmethionine.
