# Supplementary material for: Folate-Dependent Cognitive Impairment Associated With Specific Gene Networks in the Adult Mouse Hippocampus
Source: Front Nutr. 2020 Nov 12;7:574730. doi: 10.3389/fnut.2020.574730 (PMC7689186; doi:10.3389/fnut.2020.574730)
Supplement: Supplementary file 1 [file Table_1.DOCX]

**Supporting Information**

**Supplemental Figure 1. Determining daily water consumption.** In order to determine the daily water consumption per mouse per day, water bottles were filled and weighed before placing them in the cage. The average daily water consumption per mouse per day for all 12 cages (black bars) was calculated to be 5.6 + 0.4 mL (SEM) as shown by the grey dotted line.


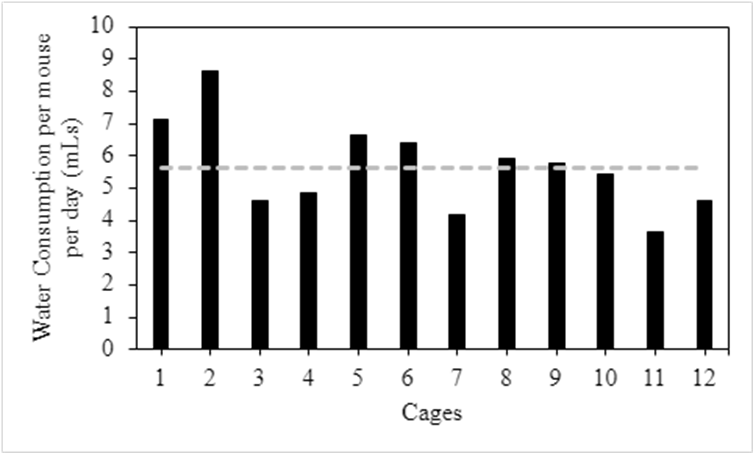


**Supplemental Figure 2. Expression of hippocampus-regulated genes in other tissues.**

Each of the genes confirmed to be differentially expressed in hippocampus were also tested in liver tissue of the same mice to determine if differential expression was systemic or tissue specific. (A) Of the 16 genes tested, only one was found to be differentially expressed in the liver (P = 0.04), and (B) none were differentially expressed in the heart. Cacna1g was omitted from the liver graph as the gene was not expressed in the liver. Error bars represent SEM values calculated from the 3-fold values prepared from three independent biological replicates.

**A**

**

**

**B**

**

**

**Supplemental Table 1. Envigo Teklad Custom folate, B_6_, and B_12_ dietary chow**. A custom dietary chow deficient in folate, B_6_, and B_12_ was obtained from Envigo Teklad Diets (Madison, WI). Methionine levels in the diet were normal. Components of the diet are listed in the table. This chowed contained the following composition by percent weight: protein 17.9%, carbohydrate 61.8%, and fat 7.0%.

| Formula | g/Kg |
| --- | --- |
| Casein, Vitamin-Free” Test | 195.0 |
| DL-methionine | 3.0 |
| Sucrose | 340.267 |
| Corn Starch | 300.0 |
| Soybean Oil | 70.0 |
| Cellulose | 40.0 |
| Mineral Mix, AIN-93G-MX (94046) | 35.0 |
| Calcium Phosphate, dibasic | 4.0 |
| TBHQ, antioxidant | 0.014 |
| Choline Bitartrate | 2.5 |
| Niacin | 0.03 |
| Calcium Pantothenate | 0.016 |
| Thiamin (81%) | 0.006 |
| Riboflavin | 0.006 |
| Biotin | 0.0002 |
| Vitamin E, DL-alpha tocopheryl acetate (500 IU/g) | 0.15 |
| Vitamin A Palmitate (500,000 IU/g) | 0.008 |
| Vitamin D_3_, choleclciferol (500,000 IU/g) | 0.002 |
| Vitamin K_1_, phylloquinone | 0.0008 |
| Succinylsulfathiazole | 10.0 |

**Supplemental Table 2. qPCR primers for gene expression.** UCSC genome browser was used to BLAST array probes and align primers over probe regions to ensure primers amplified the same transcripts as the probes. Primer3 was then used to design primers around the probe region using the following parameters where possible: GC clamps, Tm 60+2, and GC content 50+10%.

| **Gene name** | **Forward primer** | **Reverse Primer** |
| --- | --- | --- |
| Pgs1 | CTCCTGGACTTCACCAGAGG | CACCACTCAGGATGACGTTG |
| Cyp2c29 | GCATGGGTATGAAGCAGTGA | TTTTTCAGCCATTGGAAAGC |
| Izumo3 | CCTTTCATGGGGTCAGAGG | TACCAGGTTTCCCAACAAGG |
| 1700030k09Rik | GGGGACACAGTCTCCATCAC | TTGATGCATCCCATCTTTGA |
| Gtf2ird1 | TACACGCCTGGGATCCTAAC | ACACCCCAAAAACCAACAAC |
| Pacrgl | TCTGAGGGAGACCAAGCATC | AGTGCGCTCAGTCCTCTTTC |
| Lrrc34 | TTGCACTTTCCCAGTCAATG | CGTCCACCATATACGGTTCC |
| Cacna1g | TCTGCTGTGCCTTCTTCATCAT | TCAGCACAGTCGGACTTGTTAG |
| Grk4 | TGAAGCAGAATTCTCCCTCTCA | GGCTGGCATTTGTGTTCTCTTA |
| Gsta1 | CCCCAATGTGAAGAAGTTCC | TTGAAAGCCTTCCTTGCTTC |
| Rasgrp1 | CTTCGAAGCCACCAGTTGTAG | ATCCTTTGGACGTGTTTGCT |
| Map2 | ATGAAGGAAAGGCACCACAC | CCTGGTCCTTCATCTCTGGA |
| B9d1 | ATCACCGGGCAGGTAGAGA | CAGTCCTGGCCATACACAAA |
| Tomm40 | GCCAGTGAGAGCCGAGTC | CAGTGCTTGACCCAGTGC |
| Ak198376 | GGTCTCTTGCCTTGAGATGG | CCCCTGCCTACTCTCTTCAA |
| Rpl7l1 | GGGGCCTATGGAGAAGTAGC | TTAACCACTGAGCCCACCTT |

**Supplemental Table 3. List of all differentially regulated genes associated with folate deficiency.** Microarray analysis determined a total of 363 genes were downregulated and 101 genes were upregulated (P < 0.05 and fold change > 2) in response to 4 mo of post-weaning folate deficiency. Genes whose differential regulation was confirmed by qPCR analysis are noted in bold with stars.

| **GeneSymbol** | **ProbeName** | **P-value** | **Fold Change** | **Regulation** |
| --- | --- | --- | --- | --- |
| 1110008P14Rik | ASMM10P054996 | 0.01048 | 3.3046107 | Down |
| 1110008P14Rik | ASMM10P054996 | 0.01048 | 3.3046107 | Down |
| 1110008P14Rik | ASMM10P054996 | 0.01048 | 3.3046107 | Down |
| 1110008P14Rik | ASMM10P054996 | 0.01048 | 3.3046107 | Down |
| 1110008P14Rik | ASMM10P054996 | 0.01048 | 3.3046107 | Down |
| 1700006J14Rik | ASMM10P046084 | 0.04317 | 9.105104 | Down |
| 1700015E13Rik | ASMM10P024126 | 0.0006 | 2.0103375 | Down |
| 1700019L03Rik | ASMM10P020434 | 0.03247 | 2.4200944 | Down |
| **1700030K09Rik *** | ASMM10P053352 | 0.00372 | 23.4596259 | Down |
| 1700084E18Rik | ASMM10P020383 | 0.00136 | 2.2702766 | Down |
| 1810021B22Rik | ASMM10P003285 | 0.0472 | 2.3633972 | Down |
| 2010001M06Rik | ASMM10P031154 | 0.00093 | 2.1787576 | Down |
| 2010001M06Rik | ASMM10P031154 | 0.00093 | 2.1787576 | Down |
| 2310034O05Rik | ASMM10P032067 | 0.04058 | 2.4743416 | Up |
| 2310075C17Rik | ASMM10P043419 | 0.01983 | 2.5859254 | Up |
| 2310075C17Rik | ASMM10P043419 | 0.01983 | 2.5859254 | Up |
| 4833422C13Rik | ASMM10P052085 | 0.01546 | 2.1808174 | Up |
| 4930431A04Rik | ASMM10P033855 | 0.00622 | 2.4929548 | Down |
| 4930447J18Rik | ASMM10P011844 | 0.02962 | 2.0294652 | Down |
| 4930448H16Rik | ASMM10P032672 | 0.01409 | 2.0187285 | Down |
| 4930467D21Rik | ASMM10P032046 | 0.04269 | 2.4038096 | Up |
| 4930469G21Rik | ASMM10P051790 | 0.03909 | 4.8467678 | Down |
| 4930500J02Rik | ASMM10P023423 | 0.02619 | 2.2910058 | Up |
| 4930505A04Rik | ASMM10P004709 | 0.01878 | 5.7549314 | Down |
| 4930539M17Rik | ASMM10P025612 | 0.04615 | 2.5647293 | Down |
| 4930579G24Rik | ASMM10P025984 | 0.04987 | 5.7472306 | Down |
| 4933402D24Rik | ASMM10P036783 | 0.00287 | 2.0355076 | Up |
| 4933406K04Rik | ASMM10P050419 | 0.01332 | 2.0452152 | Down |
| 5530601H04Rik | ASMM10P053697 | 0.01091 | 2.0520101 | Down |
| 9330020H09Rik | ASMM10P013903 | 0.02672 | 2.0393113 | Down |
| Abca8a | ASMM10P047770 | 0.02335 | 3.4495126 | Down |
| Ablim1 | ASMM10P019378 | 0.03282 | 3.6646748 | Down |
| Actg2 | ASMM10P052880 | 0.00077 | 3.0692819 | Down |
| Adamts6 | ASMM10P010541 | 0.01015 | 2.000858 | Up |
| Adra1b | ASMM10P054016 | 0.01393 | 2.832273 | Down |
| Ahdc1 | ASMM10P003126 | 0.02362 | 2.8588009 | Down |
| Aim2 | ASMM10P024787 | 0.04299 | 8.7086599 | Down |
| AK003840 | ASMM10P017656 | 0.01476 | 2.3999598 | Up |
| AK006673 | ASMM10P032721 | 0.00531 | 2.9149948 | Down |
| AK006709 | ASMM10P040831 | 0.01645 | 4.6243684 | Down |
| AK013270 | ASMM10P027470 | 0.04486 | 2.0387172 | Down |
| AK013369 | ASMM10P024432 | 0.00405 | 2.2057914 | Down |
| AK013659 | ASMM10P025661 | 0.0464 | 3.4384463 | Up |
| AK018082 | ASMM10P035223 | 0.03593 | 7.8613069 | Down |
| AK019720 | ASMM10P014022 | 0.04621 | 3.0614789 | Down |
| AK021346 | ASMM10P034144 | 0.00771 | 2.0547582 | Down |
| AK031173 | ASMM10P040423 | 0.01223 | 2.5477543 | Down |
| AK031173 | ASMM10P040423 | 0.01223 | 2.5477543 | Down |
| AK035161 | ASMM10P045398 | 0.00553 | 2.0819174 | Down |
| AK037624 | ASMM10P032853 | 0.0081 | 6.5323606 | Down |
| AK038398 | ASMM10P039238 | 0.02888 | 3.0143524 | Up |
| AK040264 | ASMM10P019110 | 0.01614 | 2.1630015 | Down |
| AK040794 | ASMM10P049105 | 0.01477 | 4.3339645 | Down |
| AK041438 | ASMM10P020592 | 0.04409 | 8.5606078 | Down |
| AK043267 | ASMM10P010410 | 0.00788 | 2.0620991 | Down |
| AK043267 | ASMM10P010410 | 0.00788 | 2.0620991 | Down |
| AK043852 | ASMM10P049581 | 0.00793 | 2.0635063 | Up |
| AK043852 | ASMM10P049581 | 0.00793 | 2.0635063 | Up |
| AK045054 | ASMM10P029269 | 8.8E-05 | 2.2287364 | Down |
| AK047075 | ASMM10P024703 | 0.02708 | 2.4380179 | Up |
| AK047145 | ASMM10P011787 | 0.03511 | 2.228033 | Up |
| AK047272 | ASMM10P056694 | 0.02786 | 7.8993392 | Down |
| AK048114 | ASMM10P047298 | 0.00021 | 2.0531953 | Down |
| AK050631 | ASMM10P032885 | 0.00617 | 2.0977848 | Down |
| AK051019 | ASMM10P043297 | 0.00245 | 2.5146666 | Up |
| AK052812 | ASMM10P019941 | 0.02186 | 2.5686827 | Up |
| AK079717 | ASMM10P013041 | 0.02931 | 2.4259184 | Up |
| AK080592 | ASMM10P035115 | 0.0021 | 2.3311289 | Down |
| AK080611 | ASMM10P047171 | 0.03495 | 6.5359466 | Down |
| AK085233 | ASMM10P035053 | 0.01406 | 2.7159944 | Down |
| AK089776 | ASMM10P036204 | 0.00258 | 2.0125958 | Up |
| AK131777 | ASMM10P032482 | 0.00221 | 3.3110646 | Down |
| AK133313 | ASMM10P052659 | 0.04922 | 2.3445602 | Down |
| AK133331 | ASMM10P046591 | 0.00375 | 8.1543503 | Down |
| AK133422 | ASMM10P052693 | 0.01922 | 3.4401499 | Down |
| AK134272 | ASMM10P040426 | 0.00307 | 2.085219 | Up |
| AK138628 | ASMM10P039583 | 0.00339 | 3.282685 | Down |
| AK149380 | ASMM10P017389 | 0.00871 | 2.3092583 | Down |
| AK155963 | ASMM10P020620 | 0.00411 | 2.247282 | Down |
| AK156153 | ASMM10P012582 | 0.01389 | 2.2706879 | Up |
| AK157393 | ASMM10P019050 | 0.03414 | 9.3832214 | Down |
| AK157627 | ASMM10P052755 | 0.02463 | 5.4897039 | Down |
| AK158910 | ASMM10P017343 | 0.02412 | 2.1146456 | Up |
| AK161970 | ASMM10P016237 | 0.02946 | 2.3656244 | Up |
| AK180852 | ASMM10P026201 | 0.03189 | 2.8964372 | Down |
| AK180852 | ASMM10P026201 | 0.03189 | 2.8964372 | Down |
| **AK198376 *** | ASMM10P013743 | 0.00013 | 3.3275846 | Down |
| AK213200 | ASMM10P056821 | 0.03468 | 2.2073736 | Up |
| AK213543 | ASMM10P033762 | 0.00642 | 2.2731379 | Down |
| Akap2 | ASMM10P004309 | 0.0259 | 11.4858878 | Down |
| Alad | ASMM10P027231 | 0.02375 | 2.3257821 | Down |
| Alox12e | ASMM10P046977 | 0.02257 | 3.3923056 | Up |
| Ankrd27 | ASMM10P053098 | 0.00918 | 5.8208449 | Down |
| Ap1g2 | ASMM10P011096 | 0.00533 | 2.5818692 | Down |
| Ap1g2 | ASMM10P011096 | 0.00533 | 2.5818692 | Down |
| Ap2a1 | ASMM10P036043 | 0.0301 | 2.0146604 | Down |
| Arhgef18 | ASMM10P008735 | 0.00068 | 2.3490143 | Down |
| Arih2 | ASMM10P007239 | 0.02656 | 6.5158189 | Down |
| Arntl | ASMM10P006082 | 0.00414 | 2.4004106 | Up |
| Arrb2 | ASMM10P055372 | 0.00066 | 3.4515674 | Down |
| Atp13a2 | ASMM10P029814 | 0.00306 | 2.1317122 | Down |
| Atp13a2 | ASMM10P029814 | 0.00306 | 2.1317122 | Down |
| Atp2b4 | ASMM10P049425 | 0.00313 | 2.0347893 | Up |
| Atp2b4 | ASMM10P049425 | 0.00313 | 2.0347893 | Up |
| Atp6ap1l | ASMM10P052081 | 0.00208 | 2.2157722 | Up |
| Atp9b | ASMM10P056871 | 0.0085 | 2.4888953 | Down |
| Atp9b | ASMM10P056871 | 0.0085 | 2.4888953 | Down |
| Atxn7l1 | ASMM10P050744 | 0.00637 | 2.1304509 | Down |
| AV099323 | ASMM10P023818 | 0.01765 | 2.7647549 | Down |
| B4galt2 | ASMM10P055514 | 0.00812 | 2.8615736 | Down |
| **B9d1 *** | ASMM10P054063 | 0.02048 | 7.568034 | Down |
| BC027231 | ASMM10P054549 | 0.00347 | 2.5361682 | Down |
| Bcl3 | ASMM10P035669 | 0.00977 | 5.0644145 | Down |
| Bhlhb9 | ASMM10P045647 | 0.00857 | 2.2845381 | Down |
| Blvrb | ASMM10P053044 | 0.01692 | 2.21421 | Down |
| Bptf | ASMM10P047742 | 0.02901 | 2.4154991 | Down |
| Bre | ASMM10P009571 | 0.03051 | 2.0102208 | Up |
| Brinp3 | ASMM10P022500 | 0.00093 | 2.3993528 | Up |
| C78339 | ASMM10P052708 | 0.03257 | 3.3070742 | Up |
| **Cacna1g *** | ASMM10P054310 | 0.00254 | 2.1667384 | Down |
| Cacna1h | ASMM10P005395 | 0.02656 | 3.1963155 | Down |
| Cacng4 | ASMM10P047754 | 0.04281 | 2.9296468 | Down |
| Cage1 | ASMM10P051654 | 0.00076 | 3.0240019 | Up |
| Camsap3 | ASMM10P008140 | 0.02398 | 2.1360664 | Down |
| Casc5 | ASMM10P023576 | 0.04718 | 2.1327162 | Up |
| Casp1 | ASMM10P042387 | 0.01847 | 2.1868502 | Up |
| Cbfa2t3 | ASMM10P002603 | 0.01983 | 2.0572811 | Down |
| Cbfa2t3 | ASMM10P002603 | 0.01983 | 2.0572811 | Down |
| Cbfa2t3 | ASMM10P002603 | 0.01983 | 2.0572811 | Down |
| Cbln4 | ASMM10P022088 | 0.00765 | 2.2139848 | Up |
| Ccdc136 | ASMM10P002468 | 0.01563 | 2.0916718 | Down |
| Ccdc136 | ASMM10P002468 | 0.01563 | 2.0916718 | Down |
| Ccdc147 | ASMM10P019989 | 0.02839 | 2.0135521 | Up |
| Ccl19 | ASMM10P026965 | 0.00142 | 2.3526467 | Down |
| Cd72 | ASMM10P026982 | 0.01065 | 2.1535539 | Down |
| Cd72 | ASMM10P026982 | 0.01065 | 2.1535539 | Down |
| Cd72 | ASMM10P026982 | 0.01065 | 2.1535539 | Down |
| Cd72 | ASMM10P026982 | 0.01065 | 2.1535539 | Down |
| Cdc42ep5 | ASMM10P035413 | 0.00241 | 2.9883372 | Down |
| Cdv3 | ASMM10P053540 | 0.00428 | 3.6607601 | Down |
| Celsr3 | ASMM10P043370 | 0.02762 | 2.1919442 | Down |
| Cfl1 | ASMM10P019510 | 0.00278 | 2.404007 | Down |
| Cggbp1 | ASMM10P015278 | 0.00476 | 2.3116506 | Down |
| Cgnl1 | ASMM10P041867 | 0.02813 | 5.6242593 | Down |
| Chmp3 | ASMM10P034645 | 0.00603 | 2.005835 | Up |
| Clpb | ASMM10P038420 | 0.00799 | 2.3636933 | Down |
| Clstn3 | ASMM10P033763 | 0.01505 | 2.2958492 | Down |
| Clstn3 | ASMM10P033761 | 0.00282 | 2.9534725 | Down |
| Col11a1 | ASMM10P026418 | 0.00335 | 3.4514014 | Down |
| Col22a1 | ASMM10P012666 | 0.02121 | 2.0204342 | Down |
| Cpa6 | ASMM10P033688 | 0.02527 | 2.3614179 | Up |
| Crlf2 | ASMM10P055720 | 0.00084 | 2.3307929 | Down |
| Csf1r | ASMM10P054820 | 0.03111 | 2.8807058 | Down |
| Ctc1 | ASMM10P009836 | 0.00361 | 5.15251 | Down |
| Ctc1 | ASMM10P009836 | 0.00361 | 5.15251 | Down |
| Cthrc1 | ASMM10P013409 | 0.04134 | 2.1797546 | Down |
| Ctnnbip1 | ASMM10P029951 | 0.04497 | 3.1925868 | Down |
| Ctrb1 | ASMM10P039873 | 0.00029 | 3.9549903 | Down |
| Cwh43 | ASMM10P031882 | 0.00828 | 2.4697424 | Down |
| **Cyp2c29 *** | ASMM10P019865 | 0.03101 | 9.407178 | Down |
| D230017M19Rik | ASMM10P053116 | 0.04115 | 2.6084744 | Up |
| Daglb | ASMM10P032680 | 0.01389 | 2.1699465 | Down |
| Dclk1 | ASMM10P056912 | 0.03979 | 4.2066359 | Down |
| Dclk1 | ASMM10P056912 | 0.03979 | 4.2066359 | Down |
| Dclk1 | ASMM10P056912 | 0.03979 | 4.2066359 | Down |
| Dclk1 | ASMM10P056912 | 0.03979 | 4.2066359 | Down |
| Dclk1 | ASMM10P056912 | 0.03979 | 4.2066359 | Down |
| Defa-ps16 | ASMM10P003516 | 0.00227 | 3.1412353 | Down |
| Dlg1 | ASMM10P010088 | 0.0138 | 2.0858831 | Up |
| Dlg1 | ASMM10P010088 | 0.0138 | 2.0858831 | Up |
| Dlg1 | ASMM10P010088 | 0.0138 | 2.0858831 | Up |
| Dlg1 | ASMM10P010088 | 0.0138 | 2.0858831 | Up |
| Dlg1 | ASMM10P010088 | 0.0138 | 2.0858831 | Up |
| Dlg3 | ASMM10P045409 | 0.04397 | 2.5233609 | Up |
| Dlg3 | ASMM10P045409 | 0.04397 | 2.5233609 | Up |
| Dlg3 | ASMM10P045409 | 0.04397 | 2.5233609 | Up |
| Dlg3 | ASMM10P045409 | 0.04397 | 2.5233609 | Up |
| Dmrta2 | ASMM10P029302 | 0.01934 | 2.6118487 | Down |
| Dmtn | ASMM10P011294 | 0.00093 | 2.8196511 | Down |
| Dock7 | ASMM10P055477 | 0.00948 | 4.8912821 | Down |
| DOKist4 | ASMM10P057345 | 0.03911 | 2.5230507 | Up |
| Dpp4 | ASMM10P020662 | 0.01308 | 2.4954584 | Up |
| DQ568765 | ASMM10P031380 | 0.00341 | 2.0391745 | Down |
| DQ569461 | ASMM10P040796 | 0.014 | 3.8692836 | Down |
| DQ687127 | ASMM10P050302 | 0.0132 | 4.5136943 | Down |
| DQ704418 | ASMM10P050314 | 0.00057 | 2.1897486 | Down |
| E030003E18Rik | ASMM10P019725 | 0.03447 | 4.7411093 | Down |
| Eef1g | ASMM10P019595 | 0.00931 | 2.0324056 | Down |
| Efcab8 | ASMM10P057266 | 0.02255 | 2.03993 | Down |
| Eif3f | ASMM10P038576 | 0.00159 | 2.2467036 | Down |
| Elavl4 | ASMM10P055495 | 0.00342 | 3.4492798 | Down |
| Eml1 | ASMM10P051208 | 0.02244 | 2.1514807 | Down |
| Enpp5 | ASMM10P017253 | 0.00537 | 2.7246864 | Down |
| Epm2aip1 | ASMM10P053570 | 0.00487 | 2.242905 | Down |
| Fam101a | ASMM10P032467 | 0.00049 | 2.6464441 | Down |
| Fam189b | ASMM10P026067 | 0.00099 | 3.6435566 | Down |
| Fam211a | ASMM10P046856 | 0.00629 | 2.540756 | Down |
| Fat4 | ASMM10P025809 | 0.04007 | 2.3619529 | Down |
| Fchsd1 | ASMM10P017774 | 0.00624 | 2.205208 | Down |
| Ferd3l | ASMM10P050749 | 0.02449 | 2.439867 | Down |
| Fgf14 | ASMM10P057192 | 0.00251 | 2.135638 | Up |
| Fgf14 | ASMM10P057192 | 0.00251 | 2.135638 | Up |
| Fgfr4 | ASMM10P010214 | 0.00275 | 2.4966233 | Down |
| Folr4 | ASMM10P053441 | 0.0004 | 2.3850002 | Down |
| Foxk2 | ASMM10P055435 | 0.03326 | 2.4069968 | Up |
| Foxn1 | ASMM10P006690 | 0.03564 | 2.0721547 | Down |
| Foxp4 | ASMM10P016262 | 0.03672 | 3.4240533 | Down |
| Foxp4 | ASMM10P016262 | 0.03672 | 3.4240533 | Down |
| Foxp4 | ASMM10P016262 | 0.03672 | 3.4240533 | Down |
| G630090E17Rik | ASMM10P028903 | 0.03835 | 2.3463309 | Up |
| Gadl1 | ASMM10P043449 | 0.04939 | 2.4438836 | Up |
| Gdap1l1 | ASMM10P024188 | 0.00737 | 3.4369306 | Down |
| Gfra4 | ASMM10P055144 | 0.02306 | 2.2262444 | Down |
| Gja1 | ASMM10P007025 | 0.01339 | 2.3938547 | Up |
| Glt1d1 | ASMM10P032483 | 0.03211 | 2.3964964 | Up |
| Gltscr1l | ASMM10P054747 | 0.00025 | 2.5797641 | Down |
| Gm10558 | ASMM10P038685 | 0.0019 | 2.0464629 | Down |
| Gm10558 | ASMM10P038685 | 0.0019 | 2.0464629 | Down |
| Gm12014 | ASMM10P048170 | 0.03128 | 2.1984067 | Down |
| Gm12069 | ASMM10P048247 | 0.02717 | 2.8758736 | Up |
| Gm12146 | ASMM10P046562 | 0.03145 | 2.26791 | Up |
| Gm12730 | ASMM10P029224 | 0.04941 | 4.5546007 | Down |
| Gm13031 | ASMM10P028146 | 0.00082 | 2.2143717 | Down |
| Gm13390 | ASMM10P054940 | 0.04154 | 6.7902624 | Down |
| Gm13390 | ASMM10P054940 | 0.04154 | 6.7902624 | Down |
| Gm13391 | ASMM10P022278 | 0.01301 | 3.0730366 | Up |
| Gm13415 | ASMM10P022424 | 0.00207 | 2.4365366 | Down |
| Gm13429 | ASMM10P022695 | 0.04425 | 6.035512 | Down |
| Gm13716 | ASMM10P023134 | 0.00187 | 2.2648152 | Down |
| Gm13732 | ASMM10P023167 | 0.01218 | 2.1797394 | Down |
| Gm13751 | ASMM10P036820 | 0.01693 | 2.0900154 | Up |
| Gm14378 | ASMM10P040057 | 0.00988 | 2.0195495 | Down |
| Gm14664 | ASMM10P045127 | 0.00698 | 2.0762898 | Up |
| Gm14936 | ASMM10P044377 | 0.00184 | 2.0955238 | Down |
| Gm15353 | ASMM10P039078 | 0.01866 | 2.1949795 | Up |
| Gm15917 | ASMM10P032818 | 0.03686 | 2.2676629 | Down |
| Gm15917 | ASMM10P032818 | 0.03686 | 2.2676629 | Down |
| Gm16225 | ASMM10P028033 | 0.01859 | 2.1359855 | Down |
| Gm16291 | ASMM10P013415 | 0.00706 | 2.0293078 | Down |
| Gm2464 | ASMM10P024505 | 0.02708 | 6.2027938 | Down |
| Gm2464 | ASMM10P024505 | 0.02708 | 6.2027938 | Down |
| Gm25780 | ASMM10P035276 | 0.01283 | 2.1552473 | Up |
| Gm26870 | ASMM10P041159 | 0.02686 | 2.3805764 | Down |
| Gm3238 | ASMM10P031461 | 1.2E-05 | 2.3563632 | Down |
| Gm3764 | ASMM10P026040 | 0.04601 | 2.1815302 | Up |
| Gm4779 | ASMM10P053690 | 0.00014 | 2.5661438 | Down |
| Gm5 | ASMM10P031393 | 0.02553 | 2.1096751 | Up |
| Gm5778 | ASMM10P041445 | 0.00167 | 2.4879588 | Up |
| Gm6568 | ASMM10P045826 | 4.4E-05 | 3.2357924 | Down |
| Gm7092 | ASMM10P035757 | 0.0417 | 2.9174431 | Up |
| Gm9939 | ASMM10P040323 | 0.04404 | 4.1953766 | Down |
| Gpr119 | ASMM10P043876 | 0.03454 | 3.3689511 | Down |
| Gpr88 | ASMM10P025304 | 0.00266 | 3.2510551 | Up |
| Gpx1 | ASMM10P043352 | 0.03973 | 2.1518646 | Down |
| Grid1 | ASMM10P011765 | 0.00057 | 2.9255732 | Down |
| **Grk4 *** | ASMM10P004528 | 0.00396 | 4.6177633 | Down |
| **Gsta1 *** | ASMM10P053522 | 0.04768 | 2.9337349 | Up |
| Gstcd | ASMM10P025422 | 0.03548 | 2.4157009 | Down |
| Gstt4 | ASMM10P053882 | 0.04331 | 2.0113712 | Down |
| **Gtf2ird1 *** | ASMM10P055786 | 0.00115 | 2.3887171 | Down |
| H3f3b | ASMM10P047867 | 0.00218 | 4.0738156 | Down |
| Hdac10 | ASMM10P012979 | 0.00855 | 7.3592714 | Down |
| Herc2 | ASMM10P038005 | 0.0257 | 2.3842864 | Down |
| Herc3 | ASMM10P034581 | 0.00085 | 3.5134369 | Down |
| Hmga1 | ASMM10P054649 | 0.00496 | 2.2954712 | Down |
| Homez | ASMM10P011086 | 0.02771 | 2.0602019 | Down |
| Hpn | ASMM10P053090 | 0.0002 | 3.4408842 | Down |
| Hsp90ab1 | ASMM10P016192 | 0.03142 | 2.6452988 | Down |
| humanlincRNA0324 | humanlincRNA0324-_P1 | 0.02026 | 2.94356 | Down |
| humanlincRNA0488 | ASMM10P057910 | 0.02804 | 2.4792582 | Up |
| humanlincRNA1516 | humanlincRNA1516-_P1 | 0.04898 | 4.5631992 | Down |
| humanlincRNA2050 | ASMM10P057924 | 0.0484 | 2.6940291 | Down |
| humanlincRNA2246 | humanlincRNA2246-_P1 | 0.01714 | 6.6805828 | Down |
| Id3 | ASMM10P055571 | 0.00351 | 2.1198462 | Down |
| Il15ra | ASMM10P006396 | 0.00904 | 2.3512555 | Down |
| Imp4 | ASMM10P013927 | 0.01752 | 2.1997723 | Down |
| Ipo7 | ASMM10P057027 | 0.01253 | 2.1574947 | Up |
| Itfg3 | ASMM10P015784 | 0.00026 | 3.2803384 | Down |
| Itgal | ASMM10P006281 | 0.03994 | 2.0368989 | Up |
| Itpkb | ASMM10P025306 | 0.02305 | 2.3531141 | Down |
| **Izumo3 *** | ASMM10P027387 | 0.00778 | 5.2869542 | Down |
| Jade3 | ASMM10P000868 | 0.02692 | 4.2143895 | Down |
| Kat5 | ASMM10P018826 | 0.0041 | 2.1885013 | Down |
| Kbtbd2 | ASMM10P033224 | 0.00149 | 2.8199202 | Down |
| Kctd6 | ASMM10P011536 | 0.0336 | 6.3487578 | Down |
| Kif2b | ASMM10P054270 | 0.01746 | 2.4363073 | Down |
| Klf1 | ASMM10P040696 | 0.03487 | 2.1699731 | Down |
| Krt81 | ASMM10P013153 | 0.00407 | 2.5128924 | Down |
| Lamc2 | ASMM10P050821 | 0.04789 | 2.1693988 | Up |
| Lce1l | ASMM10P025016 | 0.01009 | 4.5801748 | Down |
| Lclat1 | ASMM10P017468 | 0.00119 | 2.1794352 | Down |
| Lhx6 | ASMM10P055013 | 0.00279 | 2.6490324 | Down |
| Lhx6 | ASMM10P020494 | 0.02397 | 3.7486695 | Down |
| Lpcat4 | ASMM10P023504 | 0.00633 | 3.5367943 | Down |
| Lrrc23 | ASMM10P033773 | 0.02613 | 2.0296596 | Down |
| Ltc4s | ASMM10P046631 | 0.03176 | 2.097307 | Down |
| Lzts2 | ASMM10P054921 | 0.00439 | 4.2290611 | Down |
| M19910 | ASMM10P034630 | 0.0121 | 2.483587 | Down |
| Madd | ASMM10P021077 | 0.01394 | 2.1090315 | Down |
| **Map2 *** | ASMM10P016557 | 0.00385 | 7.8245275 | Down |
| Map2k7 | ASMM10P000713 | 0.01629 | 2.2754359 | Down |
| Mapk10 | ASMM10P055706 | 0.00778 | 7.9738292 | Down |
| Mapk8ip1 | ASMM10P021106 | 0.03747 | 2.606133 | Down |
| Mast2 | ASMM10P006747 | 0.03738 | 2.8522384 | Down |
| Mat2b | ASMM10P046552 | 0.01177 | 2.6648885 | Up |
| Mat2b | ASMM10P046552 | 0.01177 | 2.6648885 | Up |
| Mcoln1 | ASMM10P040034 | 0.00179 | 2.3916513 | Down |
| Meg3 | ASMM10P051224 | 0.00616 | 2.076267 | Down |
| Metap1d | ASMM10P002081 | 0.04723 | 2.6159832 | Down |
| Mettl23 | ASMM10P055182 | 0.04908 | 4.4064578 | Down |
| Mfsd9 | ASMM10P052799 | 0.03054 | 2.0121717 | Down |
| Mllt10 | ASMM10P022359 | 0.00413 | 2.1676893 | Down |
| mouselincRNA0543 | mouselincRNA0543+_P1 | 0.0046 | 2.0289505 | Down |
| mouselincRNA0710 | ASMM10P058039 | 0.02189 | 2.8377907 | Down |
| mouselincRNA1097 | MM9LINCRNAEXON10664+_P1 | 0.00207 | 2.7130237 | Down |
| mouselincRNA1197 | MM9LINCRNAEXON10569-_P1 | 0.01841 | 2.5988611 | Down |
| mouselincRNA1298 | mouselincRNA1298-_P1 | 0.00383 | 2.5602178 | Down |
| mouselincRNA1437 | mouselincRNA1437+_P1 | 0.01035 | 2.0504115 | Down |
| mouselincRNA1592 | mouselincRNA1592+_P1 | 0.00623 | 2.0560223 | Down |
| mouselincRNA1658 | mouselincRNA1658-_P1 | 0.00568 | 2.3834295 | Up |
| mouselincRNA1671 | mouselincRNA1671-_P1 | 0.02159 | 2.0230409 | Down |
| Mpp7 | ASMM10P017602 | 0.00641 | 2.1038604 | Down |
| Mpp7 | ASMM10P017602 | 0.00641 | 2.1038604 | Down |
| Mrps27 | ASMM10P010515 | 0.01934 | 3.7004223 | Down |
| Msx3 | ASMM10P007105 | 0.00223 | 2.3482288 | Down |
| Mtag2 | ASMM10P037913 | 0.00651 | 7.9787964 | Down |
| Ndrg4 | ASMM10P053388 | 0.00454 | 3.4018637 | Down |
| Ndrg4 | ASMM10P053388 | 0.00454 | 3.4018637 | Down |
| Ndufs7 | ASMM10P042984 | 0.00488 | 2.2421574 | Down |
| Nhlrc4 | ASMM10P054642 | 0.00153 | 3.2279243 | Down |
| Ninj1 | ASMM10P052728 | 0.02855 | 2.8285304 | Down |
| Nlgn2 | ASMM10P054387 | 0.00111 | 2.6176834 | Down |
| Npb | ASMM10P009047 | 0.02546 | 2.3195504 | Down |
| Npepl1 | ASMM10P024357 | 0.02134 | 2.0499689 | Down |
| Npr1 | ASMM10P024989 | 0.00819 | 3.0145676 | Down |
| Nrbp1 | ASMM10P055648 | 0.00515 | 2.2655602 | Down |
| Nudt8 | ASMM10P054845 | 0.00341 | 2.0792572 | Down |
| Nup210 | ASMM10P033515 | 0.04087 | 2.1650045 | Down |
| Olfr1466 | ASMM10P019679 | 0.00335 | 2.6626209 | Down |
| Olfr314 | ASMM10P048586 | 0.03985 | 2.0463803 | Down |
| Olfr960 | ASMM10P042678 | 0.00981 | 3.6030806 | Down |
| Olfr961 | ASMM10P042679 | 0.0093 | 5.169211 | Down |
| **Pacrgl *** | ASMM10P007914 | 0.01473 | 5.1812522 | Down |
| Pcyt1b | ASMM10P045343 | 0.04584 | 3.1058495 | Down |
| Pcyt1b | ASMM10P045343 | 0.04584 | 3.1058495 | Down |
| Pde10a | ASMM10P000879 | 0.00276 | 2.2663374 | Down |
| Pdzd7 | ASMM10P019275 | 0.00095 | 3.7663924 | Down |
| Perp | ASMM10P038008 | 0.0034 | 2.4043106 | Down |
| Pex16 | ASMM10P023271 | 0.04804 | 2.1690322 | Up |
| Pfkfb1 | ASMM10P053736 | 0.02348 | 2.2156732 | Down |
| **Pgs1 *** | ASMM10P055217 | 0.0291 | 3.2281886 | Down |
| Phactr1 | ASMM10P054185 | 0.00912 | 2.0501473 | Down |
| Phf23 | ASMM10P048812 | 0.0293 | 2.6270992 | Down |
| Phyhd1 | ASMM10P054984 | 0.01179 | 2.7339772 | Down |
| Pla2g4c | ASMM10P037373 | 0.01565 | 12.7984093 | Down |
| Pogk | ASMM10P053798 | 0.00525 | 3.3106846 | Down |
| Polg | ASMM10P036368 | 0.014 | 3.0755858 | Down |
| Polr2i | ASMM10P037689 | 0.00821 | 2.1085968 | Down |
| Pou6f1 | ASMM10P013134 | 0.00049 | 3.5151817 | Down |
| Prr5 | ASMM10P013778 | 0.02396 | 2.0106792 | Down |
| Prss46 | ASMM10P043407 | 0.00011 | 2.6002115 | Down |
| Psmc3ip | ASMM10P047583 | 0.04514 | 6.6148139 | Down |
| Psmc3ip | ASMM10P047583 | 0.04514 | 6.6148139 | Down |
| Psmc3ip | ASMM10P047583 | 0.04514 | 6.6148139 | Down |
| Psmc3ip | ASMM10P047583 | 0.04514 | 6.6148139 | Down |
| Pth1r | ASMM10P042225 | 0.00261 | 2.9626893 | Down |
| Ptrh2 | ASMM10P049126 | 0.0113 | 2.1057218 | Down |
| Qtrtd1 | ASMM10P008388 | 0.0038 | 2.0178306 | Up |
| R3hdm1 | ASMM10P020769 | 0.00263 | 2.7109033 | Down |
| Rad51b | ASMM10P050953 | 0.00039 | 2.490915 | Down |
| **Rasgrp1 *** | ASMM10P021321 | 0.01519 | 3.0534453 | Down |
| Rasgrp2 | ASMM10P019546 | 0.04488 | 2.0383951 | Up |
| Rbfox2 | ASMM10P004793 | 0.04729 | 2.0983258 | Down |
| Rbm12 | ASMM10P008423 | 0.03207 | 6.280802 | Down |
| Rbpj | ASMM10P055670 | 0.00273 | 2.3611917 | Down |
| Rcsd1 | ASMM10P052329 | 0.01563 | 2.1657554 | Down |
| Rhox11 | ASMM10P043813 | 0.0344 | 2.1287832 | Down |
| Rims1 | ASMM10P050617 | 0.0309 | 2.7966307 | Down |
| Rnls | ASMM10P005086 | 0.04241 | 6.6422223 | Down |
| **Rpl7l1 *** | ASMM10P016236 | 9E-05 | 69.1928055 | Down |
| Rundc3a | ASMM10P054753 | 0.00463 | 2.2474624 | Down |
| Sec14l3 | ASMM10P048055 | 0.0088 | 3.5321882 | Up |
| Sec14l4 | ASMM10P048053 | 0.02921 | 2.4643467 | Down |
| Sez6 | ASMM10P048978 | 0.00562 | 2.0360277 | Down |
| Sfrp5 | ASMM10P019231 | 0.01344 | 2.1856252 | Down |
| Sgpl1 | ASMM10P001203 | 0.02427 | 3.9952272 | Down |
| Sh2d3c | ASMM10P022654 | 0.00059 | 2.4791897 | Down |
| Sh2d3c | ASMM10P022654 | 0.00059 | 2.4791897 | Down |
| Sh2d3c | ASMM10P022654 | 0.00059 | 2.4791897 | Down |
| Sh3bp5l | ASMM10P005215 | 0.04587 | 3.2090306 | Down |
| Shf | ASMM10P021427 | 0.00974 | 2.1212691 | Down |
| Slc1a3 | ASMM10P012393 | 0.02393 | 2.2921825 | Up |
| Slc22a7 | ASMM10P016222 | 0.04618 | 3.1192802 | Up |
| Smim1 | ASMM10P001602 | 0.00598 | 3.1203928 | Down |
| Smim1 | ASMM10P001602 | 0.00598 | 3.1203928 | Down |
| Smpd4 | ASMM10P014934 | 0.00065 | 2.1966571 | Down |
| Smyd3 | ASMM10P011267 | 0.00053 | 2.1208439 | Up |
| Snrnp70 | ASMM10P036072 | 0.00038 | 2.2959507 | Down |
| Socs3 | ASMM10P047915 | 0.01733 | 6.344449 | Down |
| Sod3 | ASMM10P031775 | 0.00694 | 2.5082337 | Down |
| Sos1 | ASMM10P016483 | 0.00784 | 2.0519364 | Down |
| Sp3 | ASMM10P020758 | 0.01394 | 2.3440083 | Down |
| Susd4 | ASMM10P025680 | 0.02006 | 2.728346 | Down |
| Syce2 | ASMM10P005642 | 0.03446 | 2.4829213 | Down |
| Syce2 | ASMM10P008242 | 0.02215 | 7.9927029 | Down |
| Taf9 | ASMM10P010518 | 0.00501 | 2.3209225 | Down |
| Taok1 | ASMM10P047136 | 0.00651 | 2.4628524 | Down |
| Tbata | ASMM10P053869 | 0.00162 | 4.0191825 | Down |
| Tbc1d30 | ASMM10P036000 | 0.04334 | 2.6306826 | Up |
| Tesc | ASMM10P032337 | 0.03206 | 2.3694173 | Down |
| Tex35 | ASMM10P053777 | 0.01066 | 2.0077537 | Down |
| Tfe3 | ASMM10P044793 | 0.00161 | 2.3179541 | Down |
| Themis2 | ASMM10P027963 | 0.02866 | 2.073801 | Down |
| Tlr7 | ASMM10P000755 | 0.00045 | 4.9972487 | Down |
| Tmc3 | ASMM10P038243 | 0.00566 | 2.3000231 | Down |
| Tmem107 | ASMM10P048757 | 0.04474 | 2.2410218 | Up |
| Tmem210 | ASMM10P002393 | 0.03777 | 2.035122 | Down |
| Tmem69 | ASMM10P027633 | 0.03207 | 2.9456618 | Up |
| Tnnt2 | ASMM10P021954 | 0.04241 | 2.1584923 | Up |
| Tnnt2 | ASMM10P053750 | 0.01864 | 2.5405638 | Down |
| **Tomm40 *** | ASMM10P053024 | 0.00024 | 3.2765058 | Down |
| Tor2a | ASMM10P010086 | 0.01205 | 2.0395496 | Down |
| Tor2a | ASMM10P010086 | 0.01205 | 2.0395496 | Down |
| uc.298 | ASMM10UP719 | 0.0135 | 2.5838377 | Down |
| uc.465 | ASMM10UP900 | 0.00136 | 2.9342626 | Down |
| Unkl | ASMM10P016838 | 0.04995 | 3.4422503 | Down |
| Vcp | ASMM10P026971 | 0.02083 | 2.0474611 | Up |
| Vdac2 | ASMM10P011602 | 0.035 | 2.316147 | Down |
| Vegfa | ASMM10P004151 | 0.00569 | 2.0132402 | Up |
| Vegfa | ASMM10P016210 | 0.0052 | 2.4970211 | Down |
| Vegfa | ASMM10P016210 | 0.0052 | 2.4970211 | Down |
| Vegfa | ASMM10P016210 | 0.0052 | 2.4970211 | Down |
| Vegfa | ASMM10P016210 | 0.0052 | 2.4970211 | Down |
| Vegfa | ASMM10P016210 | 0.0052 | 2.4970211 | Down |
| Vegfa | ASMM10P016210 | 0.0052 | 2.4970211 | Down |
| Vmn1r215 | ASMM10P052465 | 0.00302 | 2.0796425 | Down |
| Vps72 | ASMM10P026192 | 0.00097 | 2.0721179 | Up |
| Vwa7 | ASMM10P017105 | 0.00015 | 3.0753025 | Down |
| Wdr43 | ASMM10P017455 | 0.00668 | 2.1388965 | Down |
| Wdr62 | ASMM10P053070 | 0.00366 | 2.484518 | Down |
| XLOC_006283 | ASMM10P010414 | 0.02376 | 2.8574799 | Up |
| XLOC_006337 | ASMM10P010530 | 0.00715 | 2.5677933 | Down |
| XLOC_007382 | ASMM10P012249 | 0.02396 | 2.3676843 | Up |
| XLOC_008416 | ASMM10P012444 | 0.04837 | 2.0725831 | Up |
| XLOC_008654 | ASMM10P012862 | 0.02346 | 2.7392617 | Down |
| XLOC_011520 | ASMM10P017807 | 0.00043 | 2.4693746 | Down |
| XLOC_013198 | ASMM10P023467 | 0.00768 | 3.0972592 | Up |
| XLOC_013897 | ASMM10P020282 | 0.01586 | 2.2737216 | Down |
| XLOC_015767 | ASMM10P024701 | 0.0235 | 2.0082912 | Up |
| XLOC_015776 | ASMM10P056307 | 0.0055 | 2.0957608 | Up |
| XLOC_015982 | ASMM10P025042 | 0.03471 | 8.5681486 | Down |
| XLOC_019850 | ASMM10P052844 | 0.0016 | 3.1456838 | Down |
| XLOC_022465 | ASMM10P035736 | 0.00286 | 2.1575122 | Down |
| XLOC_024527 | ASMM10P056544 | 0.0369 | 4.9986335 | Down |
| Zar1l | ASMM10P031394 | 0.02023 | 3.6114997 | Up |
| Zcwpw2 | ASMM10P042281 | 0.04256 | 2.6123455 | Up |
| Zfand2b | ASMM10P053183 | 0.01901 | 2.2308235 | Down |
| Zfp462 | ASMM10P028864 | 0.0011 | 4.0472503 | Down |
| Zfp467 | ASMM10P033133 | 0.00913 | 3.051614 | Down |
| Zfp523 | ASMM10P016932 | 0.02926 | 3.8148587 | Down |
| Zfp592 | ASMM10P038209 | 0.04544 | 2.0293729 | Down |
| Zfp783 | ASMM10P002978 | 0.01571 | 2.0985188 | Down |
| Zfp871 | ASMM10P017019 | 0.01601 | 2.5529076 | Down |
| Zfp945 | ASMM10P004776 | 0.02451 | 3.4717836 | Up |

**Supplemental Table 4. Gene ontology with all genes listed.** All genes that were differentially expressed in the deficient condition compared to the folic acid condition according to microarray analysis (fold change > 2 and P<0.05) were analyzed with GeneCodis using all of the microarray probes as the background list. Two random lists of genes of the same size as the upregulated and two of the same size as the downregulated experimental lists were also run as controls for false positives in gene ontologies. Annotations are presented in this table if (a) the experimental annotation showed a greater number of genes or a better p-value than the random lists and (b) the annotations appeared to be relevant to this study. P-values were calculated by GeneCodis using Chi-square tests. Asterisks represent genes in the MAPK category with known implications in Alzheimer’s disease.

| Gene Set | Gene Ontology | Genes |
| --- | --- | --- |
| Genes Downregulated (317 genes) | Regulation of Nucleobase-Containing Compound | Kat5, Ankrd27, Zfp523, Ferd3l, Arrb2, Fgfr4, Prr5, Pth1r, Tnnt2, Cbfa2t3, Dmrta2, Foxp4, Npr1, Lhx6, Foxn1, Mllt10, Bcl3, Pogk, Hpn, Sp3, Pou6f1, Rbfox2, Homez, Gtf2ird1, Zfp462, Zfp467, Hmga1, Taf9, Bptf, Ctnnbip1, Rbpj, Sfrp5, Meg3, Dock7, Snrnp70, Klf1, Msx3, Npb, Hdac10, Ablim1, Cggbp1, Psmc3ip, Id3, Zfp592, Vegfa, Mapk8ip1, Tesc |
|  | Transcription from RNA Polymerase II Promoter | Kat5, Arrb2, Cbfa2t3, Foxp4, Foxn1, Polr2i, Mllt10, Bcl3, Sp3, Zfp462, Hmga1, Taf9, Bptf, Ctnnbip1, Rbpj, Sfrp5, Msx3, Hdac10, Ablim1, Psmc3ip, Id3, Vegfa |
|  | Intracellular Protein Kinase Cascade | Nrbp1, Arrb2, Fgfr4, Map2k7, Bcl3, Itpkb, Mapk10, Madd, Tlr7, Sfrp5, Gpx1, Csf1r, Ccl19, Cdc42ep5, Socs3, Taok1, Vegfa, Mapk8ip1 |
|  | Detection of Stimulus in Sensory Perception | Col11a1*, Arrb2*, Olfr961, Olfr960, Hpn, Olfr1466, Olfr314 |
|  | Ion Channel Activity | Mcoln1*, Cacna1g*, Tomm40*, Grid1*, Kctd6, Hpn, Vdac2*, Cacng4, Cacna1h |
|  | Gated Channel Activity | Cacna1g, Tomm40, Grid1, Kctd6, Hpn, Vdac2, Cacng4, Cacna1h |
|  | Double Stranded DNA Binding | Sp3, Foxp4, Aim2, Foxn1, Cggbp1 |
|  | MAPK Signaling Pathway | Arrb2*, Cacna1g*, Fgfr4*, Mapk10, Sos1, Map2k7, Taok1*, Mapk8ip1, Cacng4, Rasgrp1, Cacna1h* |
| Genes Upregulated (89 Genes) | Heparin Binding | Lamc2, Fgf14, Vegfa |
